# ﻿Performance of intron 7 of the *β*-fibrinogen gene for phylogenetic analysis: An example using gladiator frogs, *Boana* Gray, 1825 (Anura, Hylidae, Cophomantinae)

**DOI:** 10.3897/zookeys.1149.85627

**Published:** 2023-02-22

**Authors:** Ruth Amanda Estupiñán, Sávio Torres de Farias, Evonnildo Costa Gonçalves, Mauricio Camargo, Maria Paula Cruz Schneider

**Affiliations:** 1 Instituto Federal da Paraíba, Campus, João Pessoa, Paraíba, PB. CEP 58015-435, Brazil; 2 Programa de Pós-graduação em Ciências Biológicas (Zoologia), Departamento de Sistemática e Evolução, Universidade Federal da Paraíba, João Pessoa, PB. CEP 58051-900, Brazil; 3 Laboratório de Genetica Evolutiva – Paulo Leminski, Departamento de Biologia Molecular, Universidade Federal da Paraíba, João Pessoa, PB. CEP 58051-900, Brazil; 4 Laboratório de Tecnologia Biomolecular-LTB, Instituto de Ciencias Biológicas, Universidade Federal do Pará, Belém, PA. CEP 66075-110, Brazil; 5 Centro de Genómica e Biologia de Sistemas, Universidade Federal do Pará, Belém, PA, CEP 66075-110, Brazil

**Keywords:** Anura evolutionary rate, divergence time, gladiator frogs, indels, nuclear DNA, nucleotide substitution rate, phylogenetic hypothesis, polymorphic sites

## Abstract

*Boana*, the third largest genus of Hylinae, has cryptic morphological species. The potential applicability of *b-ﬁbrinogen intron 7 – FGBI7* is explored to propose a robust phylogeny of *Boana*. The phylogenetic potential of *FGBI7* was evaluated using maximum parsimony, MrBayes, and maximum likelihood analysis. Comparison of polymorphic sites and topologies obtained with concatenated analysis of *FGBI7* and other nuclear genes (*CXCR4*, *CXCR4*, *RHO*, *SIAH1*, *TYR*, and *28S*) allowed evaluation of the phylogenetic signal of *FGBI7*. Mean evolutionary rates were calculated using the sequences of the mitochondrial genes *ND1* and *CYTB* available for *Boana* in GenBank. Dating of *Boana* and some of its groups was performed using the RelTime method with secondary calibration. *FGBI7* analysis revealed high values at informative sites for parsimony. The absolute values of the mean evolutionary rate were higher for mitochondrial genes than for *FGBI7*. Dating of congruent *Boana* groups for *ND1*, *CYTB*, and *FGBI7* revealed closer values between mitochondrial genes and slightly different values from those of *FGBI7*. Divergence times of basal groups tended to be overestimated when mtDNA was used and were more accurate when nDNA was used. Although there is evidence of phylogenetic potential arising from concatenation of specific genes, *FGBI7* provides well-resolved independent gene trees. These results lead to a paradigm for linking data in phylogenomics that focuses on the uniqueness of species histories and ignores the multiplicities of individual gene histories.

## ﻿Introduction

Using only one type of trait, such as mitochondrial DNA (mtDNA), to detect phylogenetic relationships can lead to noise (Rubinoff and Holland 2005). Frog mitochondrial DNA (mtDNA) has high sequence evolution rates and many gene arrangements, making it difficult to find conserved regions (Zhang et al. 2013). The low mutation rates in mtDNA may also limit the ability to distinguish related species (Ballard and Whitlock 2004; Nabholz et al. 2008, 2009).

Introns in nuclear protein coding genes have several properties that make them useful for phylogenetic analyses of recently evolved vertebrates (Igea et al. 2010; Schmitz et al. 2017). Because they are flanked by conserved exons, they are easily amplified by polymerase chain reaction (PCR) in a variety of taxa that provide sites for PCR primers (Prychitko and Moore 2003). Introns evolve more slowly than mtDNA (Prychitko and Moore 1997, 2000; Johnson and Clayton 2000) and more rapidly than nuclear exon sequences (Hughes and Yeager 1997; Li 1997).

To obtain data on robust phylogenetic and temporal divergence in phylogeographic studies of frogs, nuclear intron data have often been used in conjunction with mtDNA data (Zhang et al. 2013; Lourenço et al. 2015; Faivovich et al. 2021; Pereyra et al. 2021). Rapidly evolving noncoding introns are used to resolve problematic nodes at the species, genus, and family levels (Prychitko and Moore 1997; Igea et al. 2010; Folk et al. 2015), and they show more robust and congruent phylogenetic signals than exons (Chen et al. 2017).

The small size of aligned base pairs (bp) and low genetic variability (variable site dataset) of *FGBI7* resulted in few informative traits and discordance between mtDNA and nuclear DNA (nDNA) (Gonçalves et al. 2007; Velo-Antón et al. 2008; Brunes et al. 2010, 2014; Prado et al. 2012; Maia-Carvalho et al. 2014; Menezes et al. 2016, 2020). These results are in contrast with previous studies related to *FGBI7* in amphibians. Thus, *FGBI7* is a valuable marker for assessing phylogenetic relationships at the family level and is likely suitable for phylogenetic analyses between closely related taxa that have recently diverged (Sequeira et al. 2006; Teixeira et al. 2015).

With 99 taxa, the Neotropical gladiator frogs of *Boana* Gray, 1825, constitute the third largest genus within Hylinae (Frost 2023). The phenotypically very similar species and lack of reliable diagnostic characters difficult the precise identity of *Boana*. Studies of cariology, morphology, vocalizations, and molecular characters have revealed cryptic species, new species, and changes in the classification of *Boana* groups (Caminer and Ron 2014, 2020; Duellman et al. 2016; Fouquet et al. 2016, 2021; Orrico et al. 2017; Ferro et al. 2018; Peloso et al. 2018; Pinheiro et al. 2019a; Lyra et al. 2020; Faivovich et al. 2021).

Two questions prompted us to conduct this study: 1) Is *FGBI7* a phylogenetic signal for *Boana* with more robust topologies than other nuclear genes? 2) Does *FGBI7* contribute to explaining the phylogeny of *Boana*? To answer these questions, we reconstructed the evolutionary history of *Boana* using several molecular markers, including *FGBI7*.

## ﻿Materials and methods

### ﻿Taxonomic sampling and DNA isolation

DNA samples were obtained from captured specimens and donations from herpetological collections (Appendix 1: Table A1). Samples included taxa from most known species groups of *Boana* (Pinheiro et al. 2019a; Faivovich et al. 2021).

Total DNA extraction from muscle or liver tissue was performed using the SDS -proteinase K/phenol-chloroform extraction method (Sambrook and Rusell 2001). *FGBI7* was sequenced on tissues from twenty-four *Boana* species (ingroup), three taxa of *Aplastodiscus*, one sample of *Bokermannohylacircumdata*, one sample of *Nesorohylakanaima*, and one of *Callimedusatomopterna* (outgroups). Primers 5’-CCATGACAATACACAACGGC-3’ and 5’-ACCACCATCCACCACCATC-3’ were designed based on the sequence of *Xenopuslaevis* (Roberts et al. 1995). After selecting the most conserved regions of *FGBI7*, the NCBI Primer- BLAST tool was used to design the target-specific primers (Ye et al. 2012). The amplification protocol was based on a 25-µL solution of 0.5–2.0 µL of the DNA template, 2.5 µL of 10× PCR buffer, 0.5 µL of each primer (10 pmol/µL), 0.5–1.5 µL of MgCl2, 1 µL of the dNTPs, and 0.15 µL of Ex Taq DNA polymerase. The PCR protocol included 3 min at 94 °C, 35 (or 30) cycles of 1 min at 94 °C, 1 min at 60 °C (or 59 s and 55 °C), and 1 min at 72 °C, and a final extension at 75 °C for 5 min.

### ﻿Sequencing and alignment

PCR products were sequenced using a MegaBACE automated DNA sequencer (GE Healthcare) and the DYEnamic ET dye terminator kit (GE Healthcare) according to the manufacturer’s instructions. Each sample was sequenced with both forward and reverse primers to confirm the observed mutations.

After searching available data in GenBank, we compared the phylogenetic signal of *FGBI7* with that of C-X-C motif chemokine receptor 4 (*CXCR4*), single exon of recombination activating gene 1 (*CXCR4*), exon 1 of Rhodopsin (*RHO*), seven-in-absentia homolog 1 (SIAH1), exon 1 of Tyrosinase (*TYR*), and *28S* ribosomal rDNA.

Sequence alignments were made using MAFFT version 7 (Katoh and Standley 2013). Alignments were edited using BioEdit (Hall 1999). Exon sequences were then concatenated using Sequence Matrix 1.7.8 (Vaidya et al. 2011) and subjected to various phylogenetic analysis methods using the same parameters as for individual genes. Genes were concatenated, although some sequences within the *Boana* taxa were not available. Missing bases that corresponded to unsequenced data were marked with a question mark.

To compare polymorphic sites and basic sequence statistics, *Boana* sequences were analyzed for conserved, variable, parsimony-informative, and singleton sites using MEGA X (Kumar et al. 2018). The number of sites without missing data (Pb*) was calculated for all genes by adding the conserved sites (C-S), singleton sites (S-S), and informative parsimony sites (P-I).

### ﻿Phylogenetic analysis

Each set of sequences for each marker was analyzed using maximum parsimony (**MP**), Bayesian analysis (**MB**), and maximum likelihood (**ML**). MP was performed in the TNT Willi Hennig Society Edition (Goloboff and Catalano 2016), and phylogenetic trees were constructed using the New Technology Search routine. Parameters selected included sectorial search, ratchet, drift, and tree fusing. A specific search was performed with an initial setting of 100 levels and run 100 times to define the minimum sequence length. Deletions were considered as a fifth base type.

Support for clades was tested using a jackknife procedure with a removal rate of 0.36, which is the most congruent value with bootstrapping (Farris et al. 1996), with absolute frequencies of 50 RAS + TBR per replicate for a total of 1,000 replicates. Consistency indices (CI), retention indices (RI), and rescaled consistency indices (CR) were calculated.

MB analysis of the evolutionary model were performed using MEGA X. For sequences with many gaps, the “use all sites” setting was selected (Tamura et al. 2011). Bayesian and Akaike information criteria were used to select the most appropriate nucleotide substitution model (Posada and Buckley 2004). The MB was run in MrBayes 3.2.7 (Ronquist et al. 2012), and sequences were considered as individual partitions for each model.

One run consisted of two repeated Monte Carlo Markov chains. The run was based on considering four chains, and the default settings for the state frequency priors (statefreqpr) were set as fixed (equal) and the substitution rate priors (ratepr) were set as variable. The other priors were set to default settings, and 85 million generations were performed (with a burn-in fraction of 0.25). Stabilization of the resulting parameters was assessed using Tracer version 1.7 (Rambaut et al. 2018) and Bayesian probability theory.

The ML analysis was performed with MEGA X software (Kumar et al. 2018) using the best substitution model generated with the same program. Bootstrap support values were used to estimate clade support based on 1,000 replicates. Missing data and gaps were included in the analyses using the “use all sites” commands. Tree inference options included nearest neighbor replacement and initial tree for ML with automatic configuration (default: NJ /BioNJ); system resource use, number of threads 1.

Phylogenetic trees were compared for each marker based on their topology and monophyletic groups defined for *Boana* (Faivovich et al. 2005, 2021; Pinheiro et al. 2019a). Trees were edited in Inkscape 0.48.5, FigTree V 1.4.4 (Rambaut 2016), and MEGA X.

### ﻿Mean evolutionary rates of ND1, CYTB, and FGBI7 nuclear genes

Mean evolutionary rates for *Boana* species were based on mitochondrial and informative genes such as *ND1* and *CYTB* from GenBank (Zhang et al. 2013). For phylogenetic inference ML, the sequences of each gene were submitted to the MEGA X software. Molecular dating for each tree, including the *FGBI7* obtained, was performed using the RelTime method (Tamura et al. 2018), a fast and powerful dating algorithm very similar to the results obtained by the Bayesian method (Mello et al. 2017; Mello 2018).

To establish a chronological scale for clade/lineage evolution, the divergence times established by Duellman et al. (2016) were used to calibrate the phylogenetic trees of the mitochondrial genes *ND1*, *CYTB*, and *FGBI7*. The following three divergence times were used for the *ND1* tree: I – divergence between *Aplastodiscus* and *Boana*, at 34.2 Ma; II – divergence of *Boanapulchella* group from the other *Boana* groups, at 22.6 Ma; and III – separation between *Boanapellucens* and *Boanarufitela*, at 5.30 Ma. The *FGBI7*-based phylogenetic tree was calibrated with the same divergence time of 34.2 Ma. The *CYTB*-based phylogenetic tree was calibrated with the divergence time between *Bokermannohyla* and *Aplastodiscus* + *Boana* of 36.8 Ma.

Divergence times were calibrated with a normal distribution and 95% confidence interval. Relative evolution rate values for each node were obtained using RelTime-Rate. Absolute evolution rates were obtained by dividing the relative rates by the scaling factor (ratio of absolute times/relative times) (Tamura et al. 2018). Mean evolutionary rates were calculated based on the absolute rates of all clades of *ND1*, *CYTB*, and *FGBI7*. After setting the calibration conditions, the “use all sites” option was selected to include all gaps and missing data in the branch length calculation (Mello 2018).

## ﻿Results

### ﻿FGBI7 DNA sequences

The average length of the *FGBI7* sequences examined was 478 base pairs. The sequences contained both single and multiple insertions and deletions. *FGBI7* sequences of 710 bp were recorded for *Boanaalbomarginata*, *Boanaalbopunctata*, *Boanalanciformis*, and *Boanaraniceps*. Alignment of long (710bp) and short sequences (478 bp) revealed short and larger deletions (230–438 positions). However, polymorphism was detected when comparing the long and short sequences.

### ﻿Nuclear DNA (nDNA) contribution to the phylogeny of *Boana*

In this study, new *FGBI7* sequences were generated for 24 *Boana* taxa. For comparison of singleton and parsimony informative sites, available sequences for 11 nuclear genes and two mitochondrial genes were retrieved from GenBank. The low number of available sequences for c-myc2, c-myc3, H3a, KIAA1239, and POMC for a large number of *Boana* taxa prevented their inclusion in the phylogenetic analysis of the group. *CXCR4*, *CXCR4*, *RHO*, *SIAH1*, *TYR*, and *28S* were used for the phylogenetic evaluation of *Boana* (Table 1).

**Table 1.** Comparative polymorphic sites and basic sequence statistics in *Boana*nDNA.

	*CXCR4*	*FGBI7*	*RAG1*	*RHO*	*SIAH1*	*TYR*	*28S*	C-genes	C-genes (1)	*ND1*	*Cyt b*
S	30	24	19	51	26	29	26	58	50	58	53
Pb	676	478	428	316	397	532	823	3650	1686	941	385
Pb*	675	466	428	316	397	532	786	3606	1673	941	385
C-S	497	286	368	254	358	387	691	2817	1153	445	198
S-S(%)	70(39)	85(47)	35(58)	27(44)	18(46)	53(37)	53(56)	358(46)	220(42)	42(8)	20(11)
P-I(%)	108(61)	95(53)	25(42)	35(56)	21(54)	92(63)	42(44)	424 (54)	300(58)	454(92)	167(89)
PIS(%) (100*P-I/Pb*)	16	20.39	5.84	11.08	5.29	17.29	5.34	11.76	17.93	99.51	99.56
AT (%)	50.4	60.4	55.8	54.5	51.3	51.9	42.5	51.3	53.2	59.5	59.3
CG (%)	49.6	39.6	44.2	45.5	48.7	48.1	57.5	48.7	46.8	40.5	40.7

C-genes = concatenated genes. C-genes (1) = concatenated (*TYR*, *FGBI7*, *CXCR4*) S = Species number. Pb = aligned base pairs. Pb* = Total number of sites, excluding missing data. C-S = conserved sites; S-S = singleton variable sites; P-I = parsimony-informative sites. PIS = Parsimony-informative sites excluding missing data. AT (%) = adenine-thymine frequency, CG (%) = cytosine-guanine frequency.

### ﻿Polymorphic sites

Informative singleton and parsimony sites comprised between 36% and 63% of all sites. The percentage of singleton sites was generally high for all genes. The percentage of parsimony-informative sites relative to the total number of sites, excluding missing data-Pb* for each of the compared genes, showed that the data based on *FGBI7*, *TYR*, and *CXCR4* gave more sensitive and highly informative performance (Table 1). Although the P-I percentage did not differ between concatenated genes (C-genes) and *FGBI7*, the percentage of P-I/Pb* parsimony-informative sites was higher for *FGBI7*. With the exception of *28S*, the frequency of A+T was higher than 50% for all genes, with *FGBI7* having the highest value.

### ﻿Phylogenetic hypothesis

**Figure 1.**
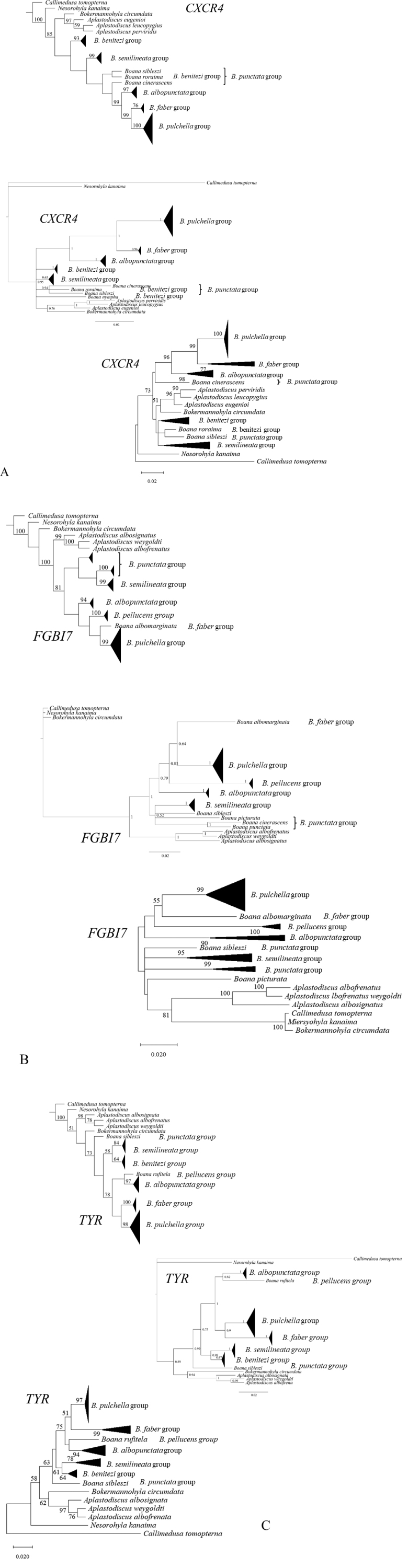
Phylogenetic trees corresponding to the studied markers provided more sensitive and highly informative performance (**A***CXCR4***B***FGBI7***C***TYR*), and the methods used–MP, MB, and ML, corresponding to the first, second, and third trees for each marker, respectively). For Jackknife support values from the MP method, and bootstrap support values for the ML method, values below 50% were not presented.

The support values of several nodes were low, ranging from 0 to 50 in all 21 trees generated by the three phylogenetic methods applied (MP, MB, and ML) (Fig. 1). Analysis of the seven nDNA markers using three different phylogenetic methods led to conflicting hypotheses about the monophyly of *Boana*. Seven groups of *Boana* were more frequently classified as monophyletic by the three methods using *TYR*, *FGBI7*, and *CXCR4*. *FGBI7* suggests monophyly of *B.pellucens* group, similar to *CXCR4*, which also supports polyphyly of *B.punctata*. All groups examined were polyphyletic for *28S* (Table 2).

**Table 2.** Monophyletic species groups recovered by the three phylogenetic methods for single and concatenated gene phylogeny.

	*CXCR4*	*FGBI7*	*CXCR4*	*RHO*	*SIAH1*	*TYR*	*28S*	*C–gene*
*Boana*	MP	MP, MB	*	*	MP, MB, ML	MP, MB, ML	*	*
*B.albopunctata* group	MP, MB, ML	MP, MB, ML	–	*	*	MP, MB, ML	*	*
*B.benitezi* group	–	–	*	*	–	MP, MB, ML	*	MP
*B.faber* group	MP, MB, ML	–	–	MP, ML	–	MP, MB, ML	*	MP, MB, ML
*B.pellucens* group	–	MP, MB, ML	–	–	–	–	–	MP, MB, ML
*B.pulchella* group	MP, MB, ML	MP, MB, ML	MP	*	MP, MB, ML	MP, MB, ML	*	
*B.punctata* group	*	*	–	*	–	–	*	*
*B.semilineata* group	MP, MB, ML	MP, MB, ML	–	MP	–	MP, MB, ML	*	MP, MB; ML

MP: monophyletic group by maximum parsimony; MB: monophyletic group by MrBayes; ML: monophyletic by maximum likelihood; *: polyphyletic groups identified by the three methods; -: absent groups or a single representative species.

The MP and consistency indices for all nDNAs analyzed were > 0.5. The CI and CR indices showed a similar trend for all nDNA, indicating a lower degree of homoplasies with an increase in their values (Table 3).

**Table 3.** Consistency and retention indices of individual and concatenated genes.

	*CXCR4*	*FGBI7*	*CXCR4*	*RHO*	*SIAH1*	*TYR*	*28S*	*C-genes*
CI	0.607	0.802	0.797	0.615	0.745	0.563	0.669	0.635
RI	0.782	0.857	0.791	0.729	0.819	0.655	0.577	0.714
CR	0.475	0.687	0.630	0.448	0.610	0.369	0.386	0.453

CI = consistency; RI = retention; CR = rescaled consistency indices

### ﻿Mean evolutionary rate

*ND1* and *CYTB* gene sequence data available for *Boana* in GenBank were obtained for 58 and 53 species, respectively. The nucleotide substitution model GTR+G+I was run with MEGA X to generate the phylogenies. The T92+G model was chosen for the phylogenetic analysis of *FGBI7*. The absolute values of the mean evolutionary rates for *ND1*, *CYTB*, and *FGBI7* were 1.235198E^-2^ ± 3.61903E^-3^, coefficient of variation – CV = 29%; 1.2796789E^-2^ ± 4.6661189E^-3^, CV = 36.4%; and 1.920083E^-3^ ± 1.07878E^-3^; CV = 56% replacement/site/million years, respectively.

**Figure 2.**
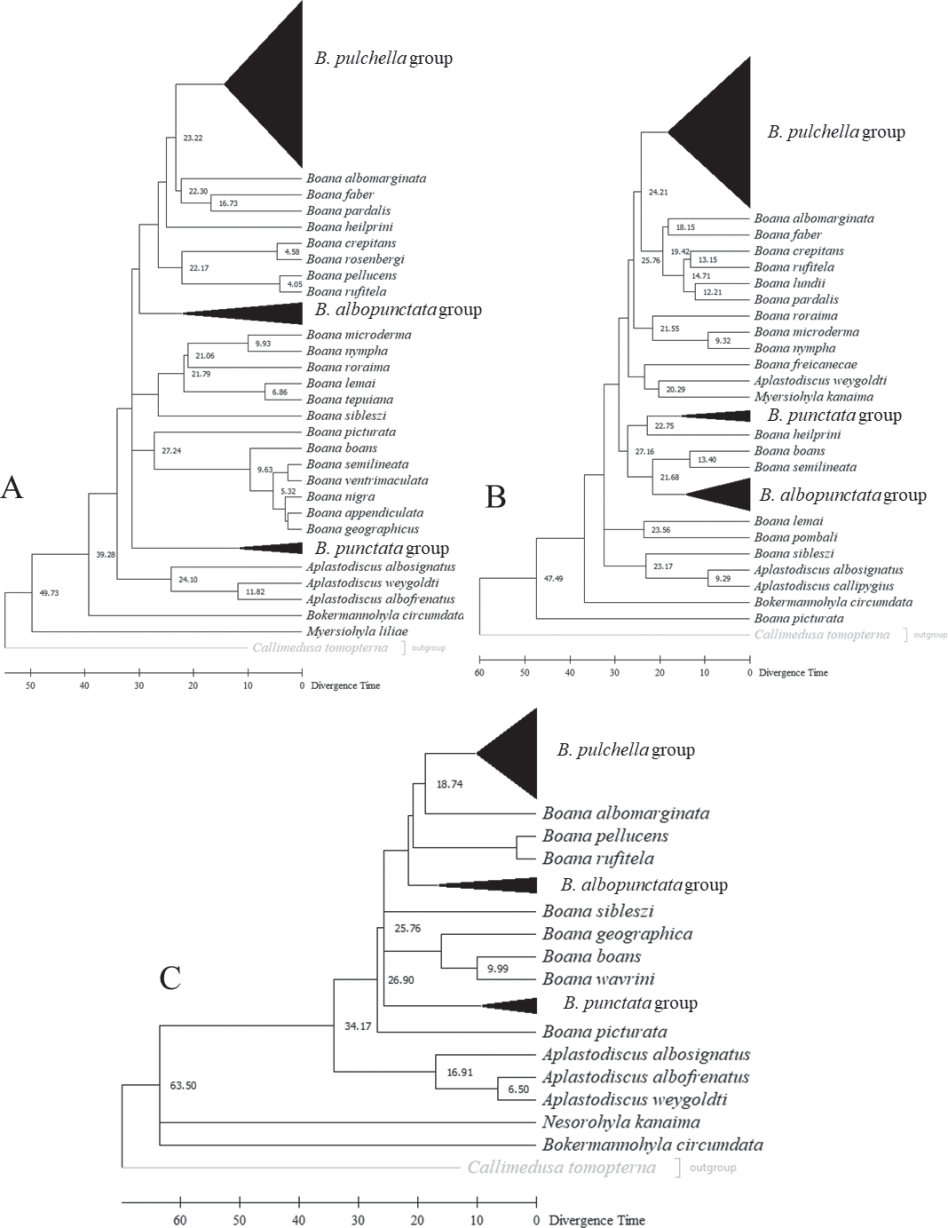
Molecular dating of gene trees **A***ND1***B***CYTB***C***FGBI7* using the RelTime method in MEGA X.

Comparison of dating results between congruent *Boana* groups for *ND1*, *CYTB*, and *FGBI7* showed divergence among the three genes, with some values being most similar among mitochondrial genes. However, the results diverged to a lesser extent from those obtained for the nuclear gene *FGBI7*. The dating results for the *B.pulchella* group revealed divergence times of 14.28 Ma (*ND1*), 15.22 Ma (*CYTB*), and 10 Ma (*FGBI7*). In addition, the *B.punctata* group (*B.cinerascens* and *B.punctata*) showed dating results of 11.44 Ma (*ND1*), 15.13 Ma (*CYTB*), and 9.12 Ma (*FGBI7*), and the *B.albopunctata* group showed divergence times of 21.94 Ma (*ND1*), 13.50 Ma (*CYTB*), and 16.32 Ma (*FGBI7*) (Fig. 2).

## ﻿Discussion

While we explored the potential applicability of *FGBI7* in reconstructing the phylogeny of *Boana* clades, our goal was to include a growing number of informative sites for future analyses, to contribute to the understanding of phylogenetic signal, and to investigate the robustness of a combination of mitochondrial and nuclear data.

Despite the short sequence (478 bp) observed in the present study, the *FGBI7*-based analyses were highly consistent with the previously proposed phylogenetic hypothesis based on the concatenation of mitochondrial and nuclear genes previously proposed for *Boana* groups (Faivovich et al. 2013, 2021; Pinheiro et al. 2019a). The phylogeny MP best agreed with these studies, showing *B.punctata* group as polyphyletic and the groups *B.semilineata* group, *B.albopunctata* group, *B.pellucens* group, *B.faber* group, and *B.pulchella* group as monophyletic.

Similar groupings were also observed among species. Some support values, such as those of *B.semilineata* group, *B.pellucens* group, and *B.pulchella* group, were very close to those determined by Pinheiro et al. (2019a).

The response of the CI and CR indices obtained by the MP analysis showed a lower degree of homoplasy for *FGBI7*. Therefore, a higher degree of parsimony compared with the congruent hypothesis generated considering the *TYR* and *CXCR4* genes and using different analysis methods supports the use of *FGBI7* in phylogenetic analysis of *Boana*.

The observed variation between the CR indices obtained for the analyzed genes can be attributed to the phylogenetic signal of indels (Miklós et al. 2004; Granados et al. 2013). Indels are a valuable source of phylogenetic information that can influence the phylogenetic outcome and have less homoplasy than nucleotides (Houde et al. 2019). The phylogenetic utility of indels may vary between individual genes; therefore, the phylogenetic weight of a single indel compared to that of a nucleotide should be explored (Pasko et al. 2011). The number, size, and distribution of indels within a sequence likely reflect the complex phenomena that lead to their accumulation over an evolutionary period and the different approaches used to analyze the available data (Houde et al. 2019).

The lowest proportion of parsimony-informative sites identified for C-genes is due to the noise of concatenation with *28S* and *RHO*. After the data for these genes were removed from the analysis (C-genes (1)), the information signal increased, although it remained lower than that of *FGBI7*. Typically, it is believed that informativeness about species history is maximized by allowing concatenation of multiple independent loci to obtain a hypothesis congruent with the species tree. However, concatenation of sequence data can bias the phylogeny if the number of gene trees that match the species tree is small. In these cases, species tree approaches can provide better-resolved phylogenies when a large number of loci are used (Edwards et al. 2007).

However, when gene tree and species tree data support a robust and congruent hypothesis (Granados et al. 2013; Ai and Kang 2015), it is possible that species trees can be resolved using only two or three few loci. These results lead to a paradigm for combining data in phylogenomics that focuses on the uniqueness of species histories and ignores the diversity of individual gene histories.

The concatenated C-gene and C-gene (1) phylogenies in MP yielded very similar clades, but the C-gene tree is not supported by bootstrap values; the C-gene (1) tree showed some high support values. The *FGBI7* tree, on the other hand, showed a larger number of bootstrap values compared to the C-gene trees (1). Tonini et al. (2015) concluded that phylogeneticists should continue to make explicit comparisons between the results of modern and classical methods.

The low reliability values obtained for multiple bootstrap and jackknife nodes and single gene trees, such as the concatenated alignment of the seven genes, indicate low robustness of the estimated topology. The low bootstrap values could be due to the small sample size and the generation of bias by signals generated by a few genes. Bootstrap values and similar support values increase with increasing numbers of sites sampled (Phillips et al. 2004).

Congruence was observed not only at the tips of trees but also at deeper inner branches. Chen et al. (2003) proposed a reliable alternative strategy in which only one bootstrap value is considered as the threshold for clade significance. In this alternative strategy, the same clade repeatedly derived from different data sets is accepted even at low bootstrap values, rather than a strongly supported clade derived from a single data set. Congruence analysis reveals different evolutionary signals in the underlying collection of genes and allows for a more conservative interpretation of phylogenomic signals (Thiergart et al. 2014). The use of *FGBI7* proved to be a complementary technique for resolving the *Boana* phylogeny. By confirming specific clades within the *Boana* phylogeny, the use of integrated traits may be better suited to elucidate the history of a clade (Salichos and Rokas 2013).

The congruence between the topology resulting from the use of *FGBI7* in this study and the results reported by Pinheiro et al. (2019a) and Faivovich et al. (2021) may be due to the design of a specific primer for *FGBI7* analysis. Other studies have also shown satisfactory results with the use of *FGBI7*-specific primers for different study groups (Sequeira et al. 2006; Teixeira et al. 2015). Designing PCR primers to screen primers against a user-selected database avoids nonspecific amplification and highlights a variable sequence of the marker to establish relationships. Although the design of specific primers is time consuming (Small et al. 2004), it has significant advantages over universal primers, particularly in terms of gene amplification, sequence quality and variation, and searching for a phylogenetic signal (Cai and Ma 2016).

Intraspecific variability in body color, description of new species, and research on declining taxa of the Hylidae (AmphibiaWeb 2021; IUCN 2021) are challenges for future studies that should be addressed using integrative trait taxonomy (Hillis 2019; Pinheiro et al. 2019b). Despite the short sequence observed in the *FGBI7*-based analysis, this is a versatile gene that can be used to address a variety of phylogenetic and taxonomic questions. The elucidation of taxa thought to be geographically widespread that are in fact cryptic species, such as *Boana* (Estupiñán et al. 2016; Fouquet et al. 2016; Caminer et al. 2017; Orrico et al. 2017; Cryer et al. 2019), and the agreement with previously proposed phylogenetic hypotheses supported by the informative sites for parsimony demonstrate the high performance of *FGBI7*.

This study also showed that *FGBI7* for *Boana* has lower mean evolutionary rates than mitochondrial genes (*ND1* and *CYTB*). The substitution rates in this study are consistent with previous reports in which nuclear genes typically had lower substitution rates than mitochondrial genes (Zheng et al. 2011; Near et al. 2012; Stöck et al. 2012). The mtDNA and nDNA evolution rates in this study were similar to those estimated by Ehl et al. (2019). These authors estimated evolution rates from E^-2^ to E^-3^ for mtDNA and from E^-3^ to E^-4^ for nDNA. However, the amplitude of evolutionary rates was lower for *FGBI7* compared with mtDNA.

The divergence dates of the *FGBI7* data were close to those obtained by Fouquet et al. (2021) for *B.punctata* group (*B.cinerascens* + *B.punctata*) (x̄ = 9.66 ± 0.76 Ma) and *B.albopunctata* (x̄ = 16.8 ± 0.70 Ma). Divergence times for *B.fasciata* were determined by Duellman et al. (2016), Feng et al. (2017), and Fouquet et al. (2021). Although Feng et al. (2017) criticized the geological dating method used by Duellman et al. (2016) and instead calibrated their dating data with fossil records, their results differed significantly from those of Fouquet et al. (2021), who used the same calibration method. Duellman et al. (2016) and Fouquet et al. (2021) found mean divergence times of 4.71 ± 1.06 Ma for *B.fasciata*, while those for *CYTB* were overestimated by 13.22 Ma.

Assuming that the divergence threshold for *Neobatrachus* from Gondwana is 145 Ma and that for Hylidae is 70 Ma, and based on nDNA data and calibration of the fossil record, the origin of *Boana* is estimated to be 25 Ma (Feng et al. 2017). Using a threshold of 62 Ma and based on nDNA and mtDNA data, Duellman et al. (2016) estimated the origin of *Boana* to be 34 Ma, with a mean diversification rate within their groups of 19.1 ± 4.6 Ma. This estimate is close to the divergence time calculated by Fouquet et al. (2021) for *B.albopunctata* (17.3 Ma) and by *FGBI7* in the present study.

Recent divergence times inferred from mtDNA sequences tend to overestimate times for basal clades (Maddin et al. 2012). On the other hand, estimates of divergence times for more recent nodes based on nuclear loci are inaccurate because significantly fewer mutations have accumulated between comparatively young lineages (Wilke et al. 2009). Another way to address this divergence is to compare these results with additional evidence. The considerable amplitude between the appearance of *Boana* is estimated to be 30 Ma and the onset of divergence of the Amazonian clade of *B.albopunctata* group is estimated to be 10 Ma. This limits our understanding of divergence times using only a single type of molecular marker. We propose that *FGBI7* should be used for anuran clades that originated between 30 and 70 Ma, while mtDNA should be used for clades that originated between 25 and 30 Ma and diverged until recently (< 2 Ma). Thus, it is possible to construct an nDNA-based time tree for a reduced set of taxa representing all genera, reconstruct different lineage-level time trees using mtDNA data, and compare the performance of the different approaches (Ehl et al. 2019).

The use of *FGBI7* in this study showed that, unlike other nuclear genes already used to generate phylogenetic hypotheses of Anura (e.g.: Wiens et al. 2010; Pyron and Wiens 2011; Duellman et al. 2016) or in the phylogenies of *Boana* and some of their groups (Faivovich et al. 2013, 2021; Caminer and Ron 2014; Pinheiro et al. 2019a; Lyra et al. 2020), has great potential to reveal relationships between lineages of very close clades.

The topology, total informative sites, and parsimony sites of *FGBI7*, in combination with mitochondrial genes, allow the clarification of new lineages already proposed by other authors such as Fouquet et al. (2021), Vasconcellos et al. (2021), and Rainha et al. (2021), contributing to future studies on *Boana* evolution and systematics.

Although estimating divergence times for clades is a difficult task (Mello 2018), the approach proposed in this study estimated the average evolutionary rate for *Boana* using two mitochondrial genes and *FGBI7*. Therefore, we recommend the use of *FGBI7* for the analysis of clades such as *Boana* with temporal ranges between 30 and 70 Ma and the use of the mtDNA genes for lineages with thresholds from the origin of *Boana*, between 25 and 30 Ma, to recent times (< 2 Ma).

## ﻿Appendix 1

**Table A1.** Voucher information, localities, and GenBank accession numbers for the sequences analyzed for this study.

Species	*CXCR4*	*Intron 7*	*RAG-1*	*RHO*	*SIAH1*	Tyr	*28S*	Voucher and source literature of sequences
*Boanaaguilari*	MT824211			MT824337			KF751464	Faivovich et al. (2013, 2021)
*Boanaalbomarginata*	KF751476	OQ448590	AY844384	AY844568	AY844794		AY844218	MRT 5870: Jussara, Bahia, Brazil. Faivovich et al. (2005, 2013)
*Boanaalbopunctata*		OQ448612		AY844569	AY844795	AY844041		MRT 8229, Petrolina, Goiás, Brazil. Faivovich et al. (2005)
*Boanabalzani*	MT824213	OQ448599	AY844395	AY844582	AY844806		AY844226	MNCN/ADN 5785, Camino a San Onofre, Carrasco, Cochabamba, Bolivia. Faivovich et al. (2005, 2021)
*Boanabenitezi*	KF751477		AY844396	AY844583			AY844227	Faivovich et al. (2005, 2013)
*Boanabischoffi*	MT824219	OQ448607	AY844398	MT824343		MT824526		AF 327, Fazenda Intervales, Estado de São Paulo. Faivovich et al. (2005, 2021)
*Boanaboans*	KF751478	OQ448591		AY844588	AY844809	AY844055	AY844231	MPEG 17385, Arredores da Fazenda passo Formoso, Manicoré, Amazonas. Faivovich et al. (2005, 2013)
*Boanabotumirim*				MT824344				Faivovich et al. (2021)
*Boanaburiti*		OQ448601		MT824346	MT824484			CHUNB 30653, Brasilia, Distrito Federal. Faivovich et al. (2021)
*Boanacaingua*	KF751479	OQ448602		MT824352	AY844812	AY844057	AY844234	AF 515, Ribeirão Grande São Paulo, São Paulo. Faivovich et al. (2005, 2013, 2021)
*Boanacalcarata*							AY844235	Faivovich et al. (2005)
*Boanacambui*				MT824356	MT824486	MT824534		Faivovich et al. (2021)
*Boanacinerascens*	KF751480	OQ448595		AY844610			DQ283466	RAET 505, Estação Científica Ferreira Penna, Melgaço, Pará, Brazil. Faivovich et al. (2005, 2013)
*Boanacipoensis*				MT824357	MT824487			Faivovich et al. (2021)
*Boanacordobae*	KF751481		AY844411	MT824460	MT824516	AY844066	AY844244	Faivovich et al. (2005, 2013, 2021)
*Boanacrepitans*	KF751482			AY844601		AY844067		Faivovich et al. (2005, 2013)
*Boanacurupi*	MT824227			MT824359	MT824489			Faivovich et al. (2021)
*Boanaericae*			AY844416	AY844605		MT824537		Faivovich et al. (2005, 2021)
*Boanafaber*				AY844607				Faivovich et al. (2005)
*Boanafasciata*	KX200378			AY844608				Faivovich et al. (2005); Feng et al. (2017)
*Boanafreicanecae*	MT824217			MT824366		MT824538		Faivovich et al. (2021)
*Boanageographica*		OQ448611						QCAZ 16809: Estación Científica Yasuní. PUCE, Laguna, Orellana, Ecuador. in confirmation process
*Boanagladiator*	MT824212			MT824368				Faivovich et al. (2021)
*Boanagoiana*				MT824372	MT824491	MT824541		Faivovich et al. (2021)
*Boanaguentheri*	MT824245	OQ448608		MT824373	MT824492		AY844253	CFBH 3386: Terra de Areia, Rio Grande do Sul, Brazil. Faivovich et al. (2005, 2021)
*Boanaheilprini*				AY844613				Faivovich et al. (2005)
*Boanajaguariaivensis*				MT824374	MT824494			Faivovich et al. (2021)
*Boanajoaquini*	KF751484	OQ448605	AY844421	MT824376			AY844256	CFBH 1068: Urubici, Santa Catarina, Brazil. Faivovich et al. (2005, 2013, 2021)
*Boanalanciformis*				AY844619		AY844081	AY844258	Faivovich et al. 2005
*Boanalemai*	KF751485		AY844423	AY844620		AY844082	AY844259	Faivovich et al. (2005, 2013)
*Boanaleptolineata*	MT824246	OQ448604	AY844424	AY844621	AY844839	AY844083	AY844260	CFBH 8504: São Francisco de Paula, Rio Grande do Sul, Brazil. Faivovich et al. (2005, 2021)
*Boanalundii*				AY844623		AY844085	AY844262	Faivovich et al. (2005)
*Boanamarginata*	KF751486		AY844426	AY844624		MT824542	AY844263	Faivovich et al. (2005, 2013, 2021)
*Boanamarianitae*		OQ448610	AY844427	MT824378	AY844843			MNCN/ADN 5901: Camino a Bella Vista, Florida, Santa Cruz, Bolivia. Faivovich et al. (2005, 2021)
*Boanamelanopleura*	KF751487	OQ448600		MT824379	HM444787			MTD-TD 1146: Huancabamba, Pasco, Peru. Faivovich et al. (2013, 2021)
*Boanamultifasciata*	GQ365986		AY844436	AY844633		AY844093	AY844270	Faivovich et al. (2005, 2010)
*Boananympha*	KF751488			AY844661		AY844112	AY844289	Faivovich et al. (2005, 2013)
*Boanapardalis*				AY844637				Faivovich et al. (2005)
*Boanapellucens*		OQ448597						QCAZ 15354: Via Toachi-Chiriboga. Poza junto a carretera cerca del Rio Orito, Ecuador.
*Boanapicturata*		OQ448594						QCAZ 15549:3 Km from Durango, em el cruce de la vá San Lorenzo e outra carretera X, Esmereldas, Ecuador.
*Boanapoaju*				MT824380	MT824495			Faivovich et al. (2021)
*Boanapolytaenia*	MT824241	OQ448606	AY844443	MT824429	MT824508	MT824547		CFBH 8394: Cristina, MG. Faivovich et al. (2005, 2021)
*Boanapombali*	MT824247			MT824431	MT824511	MT824552		Faivovich et al. (2021)
*Boanaprasina*				MT824436		MT824554		Faivovich et al. (2021)
*Boanapulchella*		OQ448609	AY844445	MT824443	MT824513	MT824557	AY844278	CHUNB 37686: Pilar do Sul, Estado de São Paulo, Brazil. Faivovich et al. (2005, 2021)
*Boanapunctata*		OQ448596		AY844645				QCAZ18185: Estación Biológica Jatun Sacha, Napo. Faivovich et al. (2005)
*Boanaraniceps*	KF751489	OQ448613		AY844646	AY844863	AY844103		MRT 6706: UHE Lajeado Tocantins. Faivovich et al. (2005, 2013)
*Boanariojana*	MT824238		AY844447	MT824462	MT824518		AY844279	Faivovich et al. (2005, 2021)
*Boanaroraima*	KF751490		AY844448	AY844650		AY844104	AY844280	Faivovich et al. (2005, 2013)
*Boanarufitela*		OQ448598		AY844652	AY844867	AY844105	AY844282	CHP-STRI:5114: Quebrada Guabalito, Palmarazo, Parque Nacional General de División Omar Torrijos Herrera, Provincia de Coclé. Faivovich et al. (2005)
*Boanasemiguttata*		OQ448603	AY844452	MT824466	MT824519	MT824559	AY844285	CFBH 242: Piraquara, Paraná, Brazil. Faivovich et al. (2005, 2021)
*Boanasemilineata*	KF751491		AY844453	AY844656		AY844108	AY844286	Faivovich et al. (2005, 2013)
*Boanasibleszi*	KF751492	OQ448593	AY844455	AY844658	AY844873	AY844110	AY844288	ROM 39561: Mount Ayanganna, Guyana. Faivovich et al. (2005, 2013)
*Boanastellae*	MT824229			MT824475		MT824567		Faivovich et al. (2021)
*Boanastenocephala*				MT824479	MT824520	MT824563		Faivovich et al. (2021)
*Boanawavrini*		OQ448592						RAET 502: Estação Científica Ferreira Penna, Melgaço, Pará, Brazil
*Aplastodiscusalbofrenatus*		OQ448614	KU184083	KU184111	KU184149	KU184246		AF 101: Rio de Janeiro, Rio de Janeiro, Brazil. Berneck et al. (2016)
*Aplastodiscusalbosignatus*		OQ448616	AY844385	KU184114	AY844796	AY844042	AY844219	CFBH 7711: Parque Estadual Serra do Mar, Santa Virginia, São Luís do Paraitinga, São Paulo, Brazil. Faivovich et al. (2005)
*Aplastodiscuseugenioi*	KF751465							Faivovich et al. (2013)
*Aplastodiscusleucopygius*	KF751466						AY844261	Faivovich et al. (2005; 2013)
*Aplastodiscusperviridis*	KF751467						AY844201	Faivovich et al. (2005, 2013)
*Aplastodiscusweygoldti*		OQ448615	AY844467	KU184124	AY844887	KU184257		AF 68: São Paulo do Aracã, Espírito Santo, Brazil. Faivovich et al. (2005)
*Bokermannohylacircumdata*	KF751468	OQ448619	AY844409		AY844817	AY844064	AY844242	IT-H0562, MZUSP 93551: Juquitiba, Estado de São Paulo. Berneck et al. (2016); Faivovich et al. (2005; 2013)
*Callimedusatomopterna*	GQ366024	OQ448618	AY844497	AY844715		AY844157	AY844328	MPEG 17368, Near Fazenda Passo Formoso, Manicoré, Amazonas, Brazil. Faivovich et al. (2010, 2005)
*Callimedusavaillanti*					AY844921			Faivovich et al. (2005)
*Myersiohylaliliae*			MH251236					Pinheiro et al. (2019a)
*Myersiohylainparquesi*							AY844291	Faivovich et al. (2005)
*Nesorohylakanaima*	GQ365994	OQ448617		AY844617	MH251240	AY844079		ROM 39586: Mount Ayanganna, Guyana. Faivovich et al. (2005, 2010); Pinheiro et al. (2019a)

AF: Laboratório de Citogenética de Vertebrados. Depto. Genética e Biologia Evolutiva, Instituto de biologia, Universidade de São Paulo. CHP- STRI: Circulo Herpetologico de Panama-Smithsonian Tropical Research InstituteCFBH: Célio F. B. Haddad. Coleção de anfíbios, SP, Brazil CHUNB: Coleção herpetológica, Universidade de Brasília, BrazilMNCN: Museu Nacional de Ciências Naturales, SpainMPEG: Museu Paraense Emilio Goeldi, BrazilMRT: Miguel Trefaut Urbano Rodrigues, Universidade de São Paulo, Brazil. MTD-TD: Museum für Tierkunde, Germany. MZUSP: Museu de Zoologia, Universidade de São Paulo, BrazilQCAZ: Colección de anfibios del Museo de Zoología, Pontificia Universidad Católica del EcuadorRAET: Ruth Amanda Estupina Tristancho, Brazil ROM: Royal Ontario Museum, Centre for Biodiversity and Conservation Biology: Herpetology, Canada

## Supplementary materials

Supplementary material 1 Phylogenetic trees and molecular dating of gene trees Data type pdf file Explanation note Phylogenetic trees corresponding to the studied markers (a. CXCR4, b. FGBI7, c. RAG-1, d. RHO, e. TYRf.SIAH1, g. 28SandC-genes), and the methods used – MP, MB, and ML, corresponding to the 1^st^, 2^nd^, and 3^rd^ trees for each marker, respectively). For Jackknife support values from the MP method, and bootstrap support values for the ML method, values below 50% were not presented. Molecular dating of gene trees. a. ND1. b. CYTB, and c. FGBI7using the RelTimemethod in MEGA X. https://binary.pensoft.net/file/814496This dataset is made available under the Open Database License (http://opendatacommons.org/licenses/odbl/1.0/). The Open Database License (ODbL) is a license agreement intended to allow users to freely share, modify, and use this Dataset while maintaining this same freedom for others, provided that the original source and author(s) are credited.Ruth Amanda Estupiñán, Sávio Torres de Farias, Evonnildo Costa Gonçalves, Mauricio Camargo, Maria Paula Cruz Schneider
