# Supplementary material for: ﻿Performance of intron 7 of the β-fibrinogen gene for phylogenetic analysis: An example using gladiator frogs, Boana Gray, 1825 (Anura, Hylidae, Cophomantinae)
Source: Zookeys. 2023 Feb 22;1149:145–69. doi: 10.3897/zookeys.1149.85627 (PMC10209274; doi:10.3897/zookeys.1149.85627)
Supplement: Supplementary material 1 — Phylogenetic trees and molecular dating of gene trees [file zookeys-1149-145_article-85627__-s001.pdf]

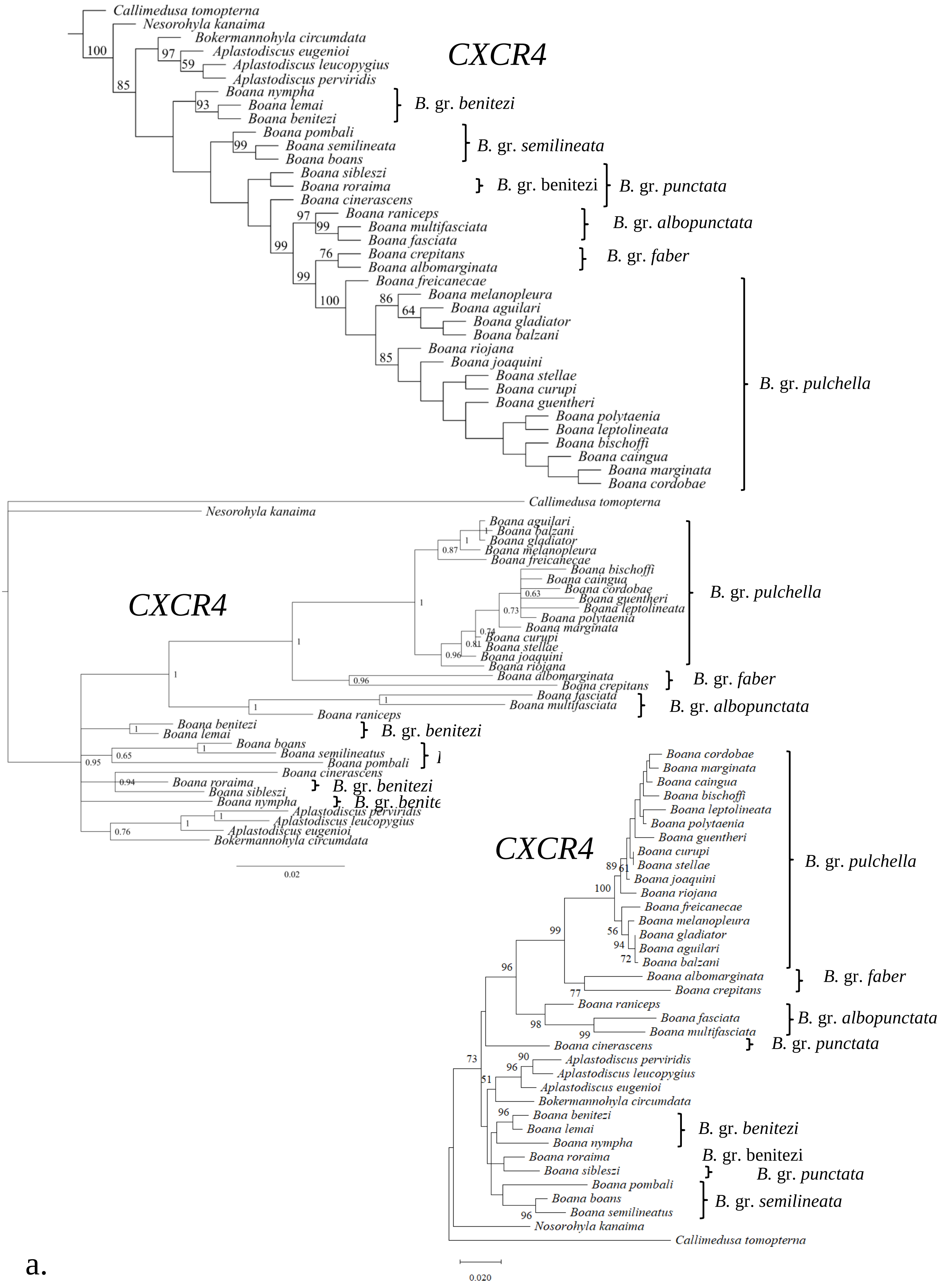
CXCR4
Callimedusa tomopterna
Nesorohyla kanaima
Bokermannohyla circumdata
Aplastodiscus eugenioi
Aplastodiscus leucopygius
Aplastodiscus perviridis
Boana nympha
Boana lemai
Boana benitezi
B. gr. benitezi
Boana pombali
Boana semilineata
Boana boans
B. gr. semilineata
Boana sibleszi
Boana roraima
B. gr. benitezi
B. gr. punctata
Boana cinerascens
Boana raniceps
Boana multifasciata
Boana fasciata
B. gr. albopunctata
Boana crepitans
Boana albomarginata
B. gr. faber
Boana freicanecae
Boana melanopleura
Boana aguilari
Boana gladiator
Boana balzani
Boana riojana
Boana joaquini
Boana stellae
Boana curupi
Boana guentheri
Boana polytaenia
Boana leptolineata
Boana bischoffi
Boana caingua
Boana marginata
Boana cordobae
B. gr. pulchella
CXCR4
Callimedusa tomopterna
Nesorohyla kanaima
B. gr. pulchella
B. gr. faber
B. gr. albopunctata
B. gr. benitezi
B. gr. benitezi
0.02
CXCR4
Boana cordobae
Boana marginata
Boana caingua
Boana bischoffi
Boana leptolineata
Boana polytaenia
Boana guentheri
Boana curupi
Boana stellae
Boana joaquini
Boana riojana
Boana freicanecae
Boana melanopleura
Boana gladiator
Boana aguilari
Boana balzani
B. gr. pulchella
Boana albomarginata
Boana crepitans
B. gr. faber
Boana raniceps
Boana fasciata
Boana multifasciata
B. gr. albopunctata
Boana cinerascens
B. gr. punctata
Aplastodiscus perviridis
Aplastodiscus leucopygius
Aplastodiscus eugenioi
Bokermannohyla circumdata
Boana benitezi
Boana lemai
Boana nympha
B. gr. benitezi
Boana roraima
Boana sibleszi
B. gr. benitezi
B. gr. punctata
Boana pombali
Boana boans
Boana semilineatus
B. gr. semilineata
Nosorohyla kanaima
Callimedusa tomopterna
0.020

a.

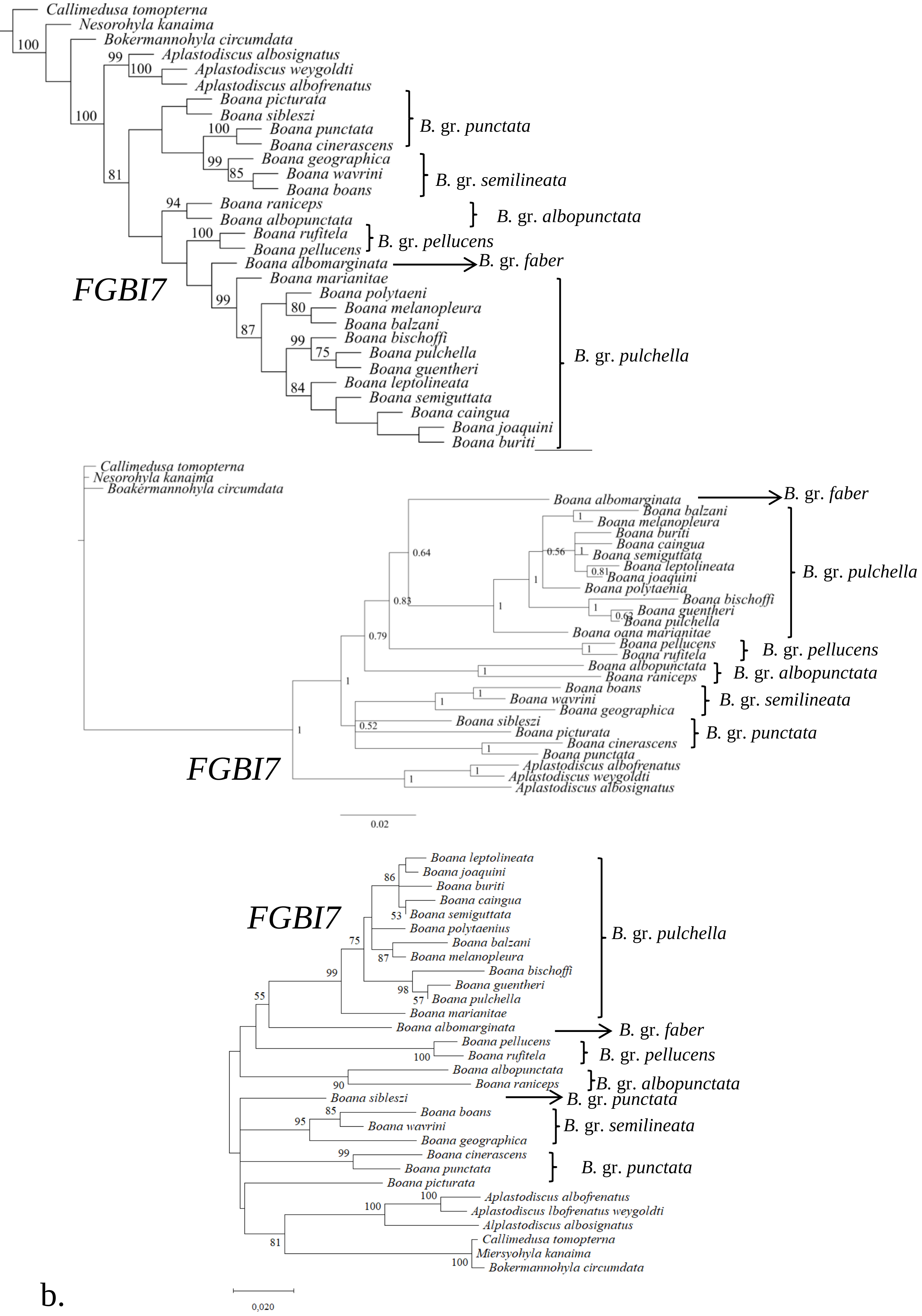

Callimedusa tomopterna
Nesorohyla kanaima
Bokermannohyla circumdata
Aplastodiscus albosignatus
Aplastodiscus weygoldti
Aplastodiscus albofrenatus
Boana picturata
Boana sibleszi
Boana punctata
Boana cinerascens
B. gr. punctata
Boana geographica
Boana wavrini
Boana boans
B. gr. semilineata
Boana raniceps
Boana albopunctata
B. gr. albopunctata
Boana rufitela
Boana pellucens
B. gr. pellucens
Boana albomarginata
B. gr. faber
FGBI7
Boana marianitae
Boana polytaeni
Boana melanopleura
Boana balzani
Boana bischoffi
Boana pulchella
Boana guentheri
Boana leptolineata
Boana semiguttata
Boana caingua
Boana joaquini
Boana buriti
B. gr. pulchella
100
99
100
100
100
99
85
81
94
100
99
87
80
99
75
84
Callimedusa tomopterna
Nesorohyla kanaima
Boakermannohyla circumdata
Boana albomarginata
B. gr. faber
Boana balzani
Boana melanopleura
Boana buriti
Boana caingua
Boana semiguttata
Boana leptolineata
Boana joaquini
Boana polytaenia
Boana bischoffi
Boana guentheri
Boana pulchella
Boana oana marianitae
B. gr. pulchella
Boana pellucens
Boana rufitela
B. gr. pellucens
Boana albopunctata
Boana raniceps
B. gr. albopunctata
Boana boans
Boana wavrini
Boana geographica
B. gr. semilineata
Boana sibleszi
Boana picturata
Boana cinerascens
Boana punctata
B. gr. punctata
Aplastodiscus albofrenatus
Aplastodiscus weygoldti
Aplastodiscus albosignatus
FGBI7
0.64
0.56
0.81
0.83
0.79
0.63
0.52
0.02
Boana leptolineata
Boana joaquini
Boana buriti
Boana caingua
Boana semiguttata
Boana polytaenius
Boana balzani
Boana melanopleura
Boana bischoffi
Boana guentheri
Boana pulchella
Boana marianitae
B. gr. pulchella
FGBI7
Boana albomarginata
B. gr. faber
Boana pellucens
Boana rufitela
B. gr. pellucens
Boana albopunctata
Boana raniceps
B. gr. albopunctata
Boana sibleszi
B. gr. punctata
Boana boans
Boana wavrini
Boana geographica
B. gr. semilineata
Boana cinerascens
Boana punctata
Boana picturata
B. gr. punctata
Aplastodiscus albofrenatus
Aplastodiscus lbofrenatus weygoldti
Alplastodiscus albosignatus
Callimedusa tomopterna
Miersyohyla kanaima
Bokermannohyla circumdata
86
53
75
87
99
98
57
55
100
90
85
95
99
100
100
81
100
0,020
b.

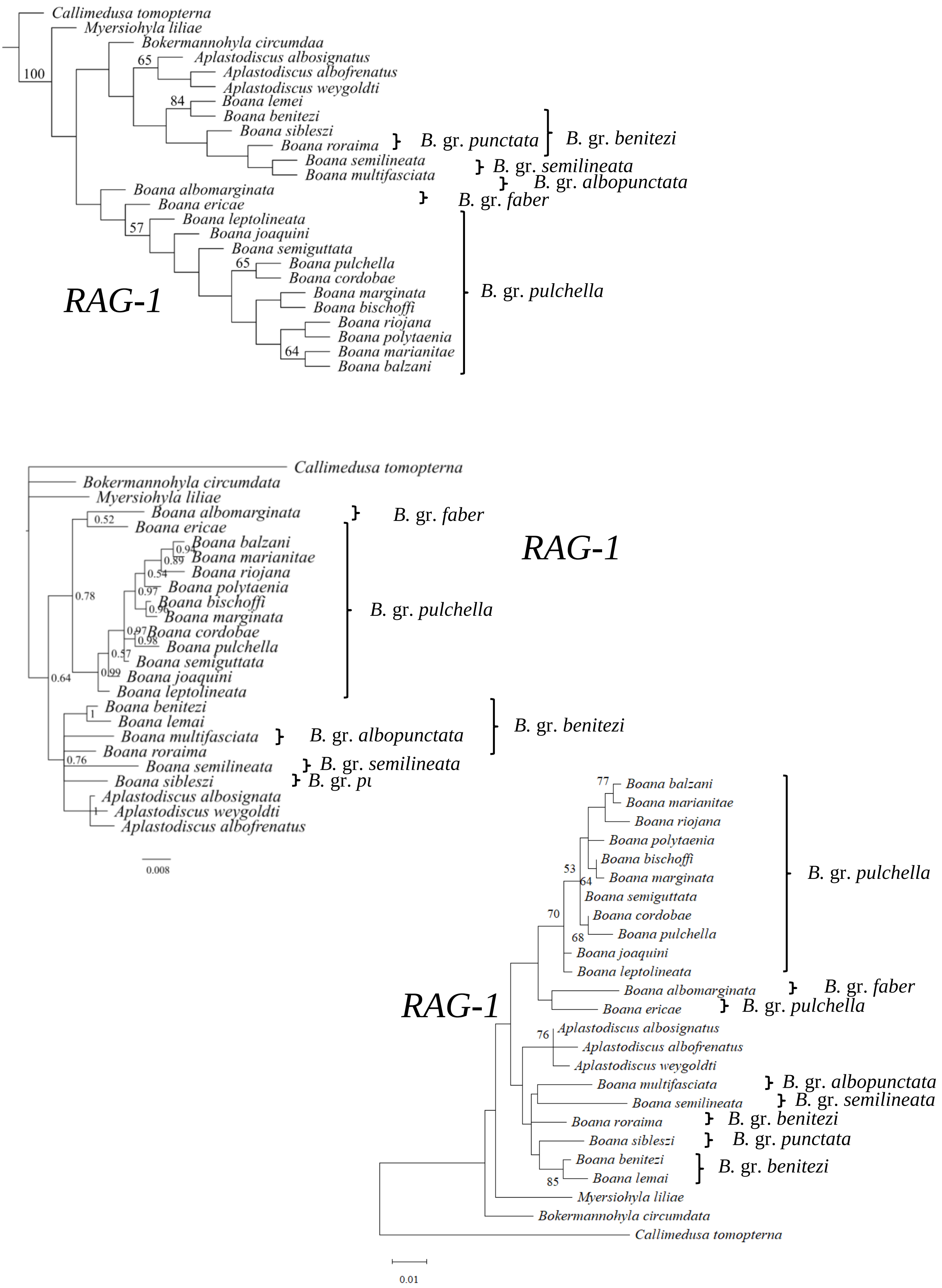
RAG-1
Callimedusa tomopterna
Myersiohyla liliae
Bokermannohyla circumdaa
Aplastodiscus albosignatus
Aplastodiscus albofrenatus
Aplastodiscus weygoldti
Boana lemei
Boana benitezi
Boana sibleszi
Boana roraima
Boana semilineata
Boana multifasciata
Boana albomarginata
Boana ericae
Boana leptolineata
Boana joaquini
Boana semiguttata
Boana pulchella
Boana cordobae
Boana marginata
Boana bischoffi
Boana riojana
Boana polytaenia
Boana marianitae
Boana balzani
100
65
84
57
65
64
B. gr. punctata
B. gr. benitezi
B. gr. semilineata
B. gr. albopunctata
B. gr. faber
B. gr. pulchella
RAG-1
Callimedusa tomopterna
Bokermannohyla circumdata
Myersiohyla liliae
Boana albomarginata
Boana ericae
Boana balzani
Boana marianitae
Boana riojana
Boana polytaenia
Boana bischoffi
Boana marginata
Boana cordobae
Boana pulchella
Boana semiguttata
Boana joaquini
Boana leptolineata
Boana benitezi
Boana lemai
Boana multifasciata
Boana roraima
Boana semilineata
Boana sibleszi
Aplastodiscus albosignata
Aplastodiscus weygoldti
Aplastodiscus albofrenatus
0.52
0.94
0.89
0.54
0.97
0.96
0.97
0.98
0.57
0.99
0.78
0.64
1
0.76
1
0.008
B. gr. faber
B. gr. pulchella
B. gr. albopunctata
B. gr. benitezi
B. gr. semilineata
B. gr. pı
RAG-1
Boana balzani
Boana marianitae
Boana riojana
Boana polytaenia
Boana bischoffi
Boana marginata
Boana semiguttata
Boana cordobae
Boana pulchella
Boana joaquini
Boana leptolineata
Boana albomarginata
Boana ericae
Aplastodiscus albosignatus
Aplastodiscus albofrenatus
Aplastodiscus weygoldti
Boana multifasciata
Boana semilineata
Boana roraima
Boana sibleszi
Boana benitezi
Boana lemai
Myersiohyla liliae
Bokermannohyla circumdata
Callimedusa tomopterna
77
53
64
70
68
76
85
0.01
B. gr. pulchella
B. gr. faber
B. gr. pulchella
B. gr. albopunctata
B. gr. semilineata
B. gr. benitezi
B. gr. punctata
B. gr. benitezi

c.

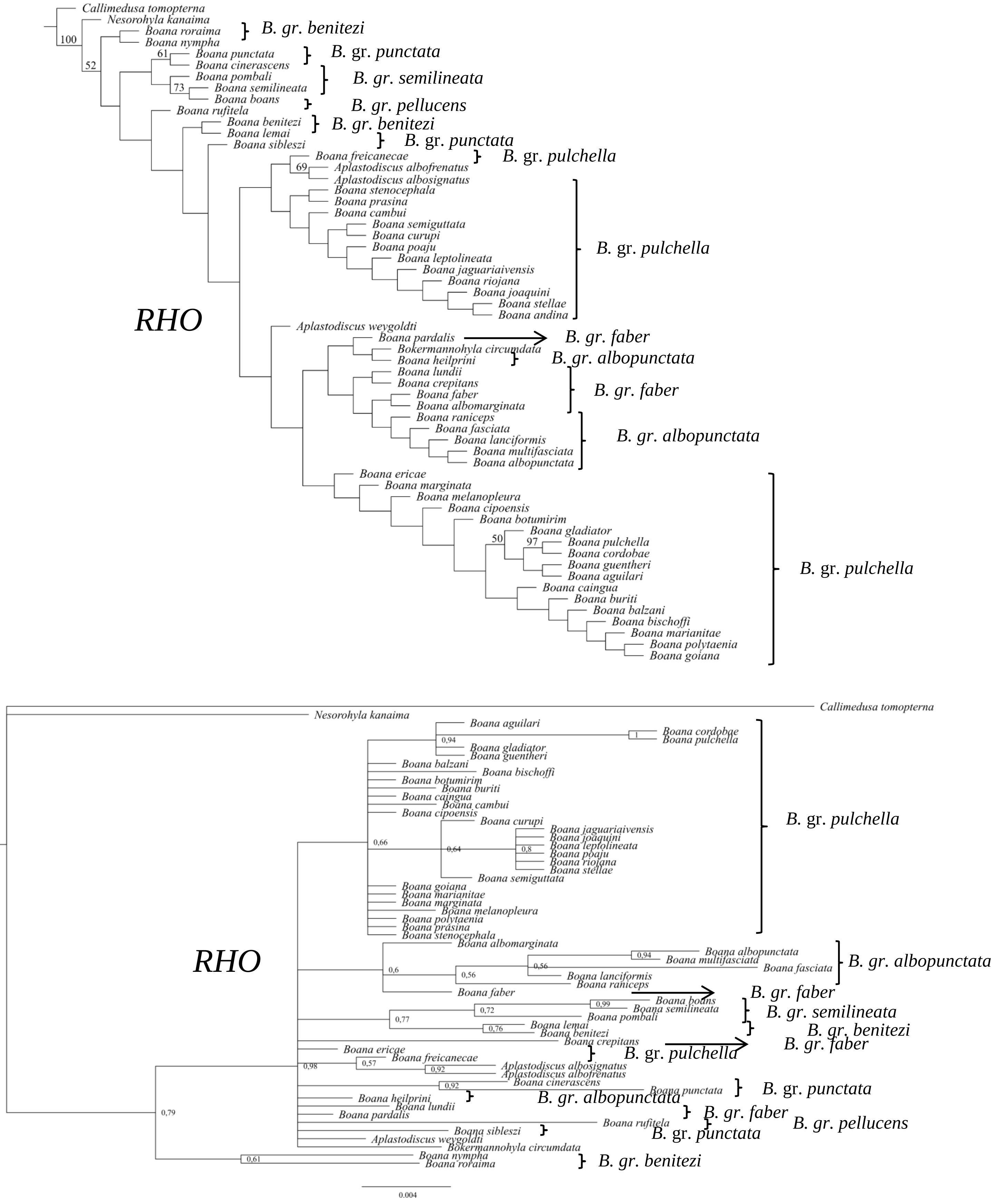

Callimedusa tomopterna
Nesorohyla kanaima
100
Boana roraima
Boana nympha
B. gr. benitezi
52
61
Boana punctata
Boana cinerascens
B. gr. punctata
Boana pombali
73
Boana semilineata
Boana boans
B. gr. semilineata
B. gr. pellucens
Boana rufitela
Boana benitezi
Boana lemai
B. gr. benitezi
Boana sibleszi
B. gr. punctata
Boana freicanecae
69
Aplastodiscus albofrenatus
Aplastodiscus albosignatus
B. gr. pulchella
Boana stenocephala
Boana prasina
Boana cambui
Boana semiguttata
Boana curupi
Boana poaju
Boana leptolineata
Boana jaguariaivensis
Boana riojana
Boana joaquini
Boana stellae
Boana andina
B. gr. pulchella
RHO
Aplastodiscus weygoldti
Boana pardalis
B. gr. faber
Bokermannohyla circumdata
Boana heilprini
B. gr. albopunctata
Boana lundii
Boana crepitans
Boana faber
Boana albomarginata
B. gr. faber
Boana raniceps
Boana fasciata
Boana lanciformis
Boana multifasciata
Boana albopunctata
B. gr. albopunctata
Boana ericae
Boana marginata
Boana melanopleura
Boana cipoensis
Boana botumirim
50
Boana gladiator
97
Boana pulchella
Boana cordobae
Boana guentheri
Boana aguilari
Boana caingua
Boana buriti
Boana balzani
Boana bischoffi
Boana marianitae
Boana polytaenia
Boana goiana
B. gr. pulchella
Callimedusa tomopterna
Nesorohyla kanaima
Boana aguilari
0,94
1
Boana cordobae
Boana pulchella
Boana gladiator
Boana guentheri
Boana balzani
Boana bischoffi
Boana botumirim
Boana buriti
Boana caingua
Boana cambui
Boana cipoensis
Boana curupi
0,66
0,64
0,8
Boana jaguariaivensis
Boana joaquini
Boana leptolineata
Boana poaju
Boana riojana
Boana stellae
Boana semiguttata
Boana goiana
Boana marianitae
Boana marginata
Boana melanopleura
Boana polytaenia
Boana prasina
Boana stenocephala
B. gr. pulchella
RHO
Boana albomarginata
0,94
Boana albopunctata
Boana multifasciata
Boana fasciata
0,6
0,56
0,56
Boana lanciformis
Boana raniceps
B. gr. albopunctata
Boana faber
B. gr. faber
0,99
Boana boans
Boana semilineata
0,72
Boana pombali
0,77
B. gr. semilineata
0,76
Boana lemai
Boana benitezi
B. gr. benitezi
Boana crepitans
B. gr. faber
Boana ericae
B. gr. pulchella
0,57
Boana freicanecae
0,98
0,92
Aplastodiscus albosignatus
Aplastodiscus albofrenatus
0,92
Boana cinerascens
Boana punctata
B. gr. punctata
Boana heilprini
B. gr. albopunctata
Boana lundii
0,79
Boana pardalis
B. gr. faber
Boana rufitela
B. gr. pellucens
Boana sibleszi
B. gr. punctata
Aplastodiscus weygoldti
Bokermannohyla circumdata
0,61
Boana nympha
Boana roraima
B. gr. benitezi
0.004

d.

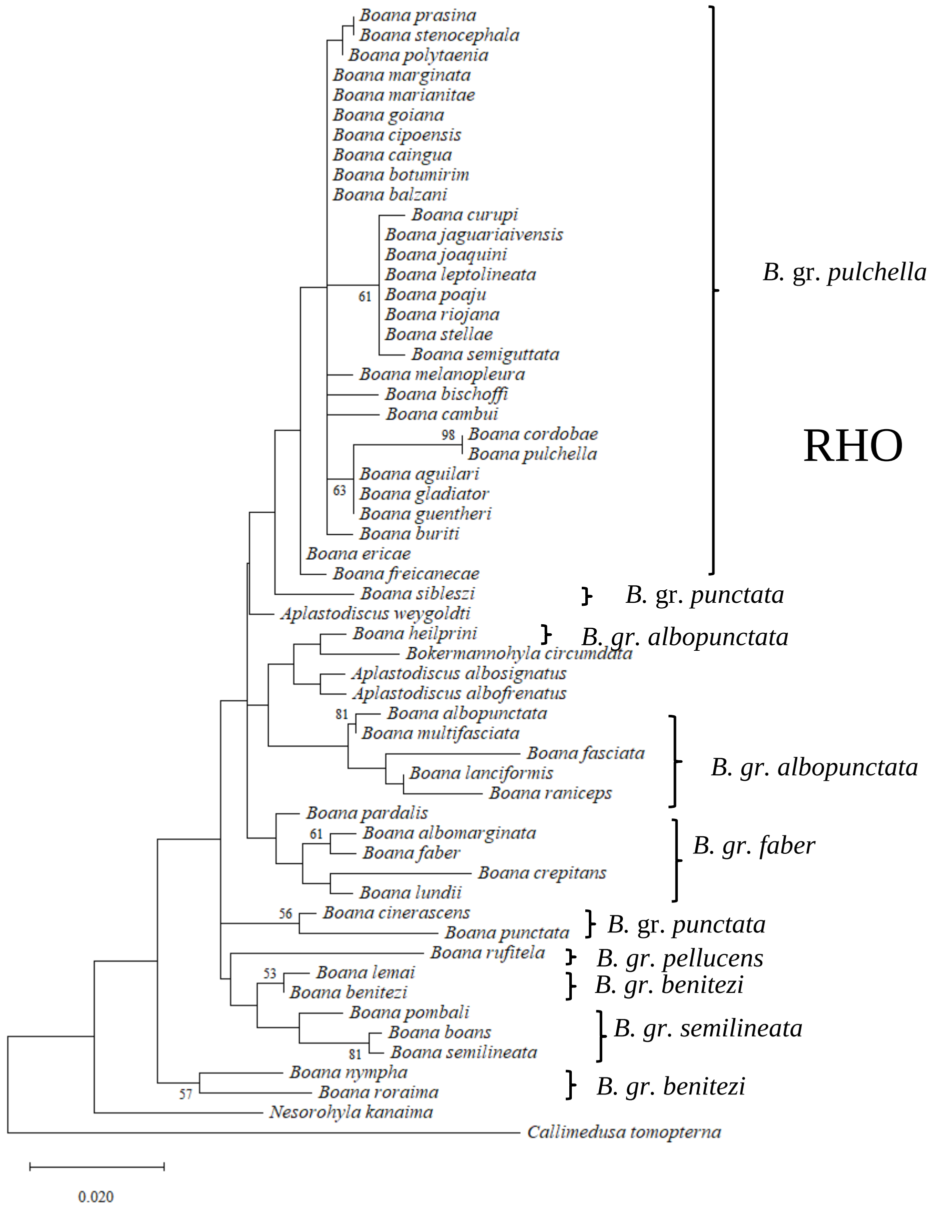
Boana prasina
Boana stenocephala
Boana polytaenia
Boana marginata
Boana marianitae
Boana goiana
Boana cipoensis
Boana caingua
Boana botumirim
Boana balzani
Boana curupi
Boana jaguariaivensis
Boana joaquini
Boana leptolineata
61
Boana poaju
Boana riojana
Boana stellae
Boana semiguttata
Boana melanopleura
Boana bischoffi
Boana cambui
98
Boana cordobae
Boana pulchella
Boana aguilari
63
Boana gladiator
Boana guentheri
Boana buriti
Boana ericae
Boana freicanecae
Boana sibleszi
Aplastodiscus weygoldti
Boana heilprini
Bokermannohyla circumdata
Aplastodiscus albosignatus
Aplastodiscus albofrenatus
81
Boana albopunctata
Boana multifasciata
Boana fasciata
Boana lanciformis
Boana raniceps
Boana pardalis
61
Boana albomarginata
Boana faber
Boana crepitans
Boana lundii
56
Boana cinerascens
Boana punctata
Boana rufitela
53
Boana lemai
Boana benitezi
Boana pombali
Boana boans
81
Boana semilineata
Boana nympha
57
Boana roraima
Nesorohyla kanaima
Callimedusa tomopterna
0.020
B. gr. pulchella
RHO
B. gr. punctata
B. gr. albopunctata
B. gr. albopunctata
B. gr. faber
B. gr. punctata
B. gr. pellucens
B. gr. benitezi
B. gr. semilineata
B. gr. benitezi

d.

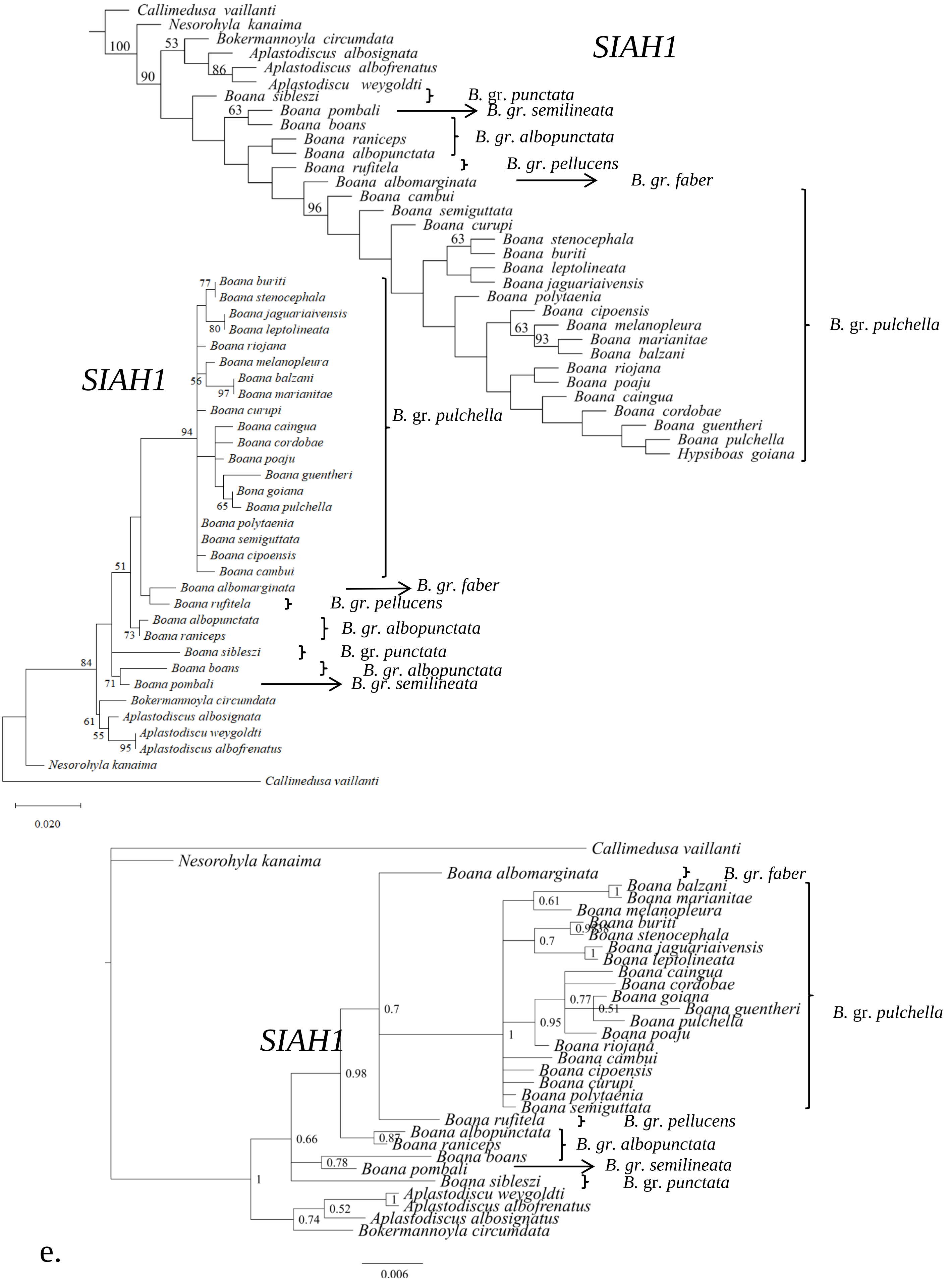
SIAH1
Callimedusa vaillanti
Nesorohyla kanaima
Bokermannoyla circumdata
Aplastodiscus albosignata
Aplastodiscus albofrenatus
Aplastodiscu weygoldti
Boana sibleszi
Boana pombali
Boana boans
Boana raniceps
Boana albopunctata
Boana rufitela
Boana albomarginata
Boana cambui
Boana semiguttata
Boana curupi
Boana stenocephala
Boana buriti
Boana leptolineata
Boana jaguariaivensis
Boana polytaenia
Boana cipoensis
Boana melanopleura
Boana marianitae
Boana balzani
Boana riojana
Boana poaju
Boana caingua
Boana cordobae
Boana guentheri
Boana pulchella
Hypsiboas goiana
100
90
53
86
63
96
63
63
93
B. gr. punctata
B. gr. semilineata
B. gr. albopunctata
B. gr. pellucens
B. gr. faber
B. gr. pulchella
SIAH1
Boana buriti
Boana stenocephala
Boana jaguariaivensis
Boana leptolineata
Boana riojana
Boana melanopleura
Boana balzani
Boana marianitae
Boana curupi
Boana caingua
Boana cordobae
Boana poaju
Boana guentheri
Bona goiana
Boana pulchella
Boana polytaenia
Boana semiguttata
Boana cipoensis
Boana cambui
Boana albomarginata
Boana rufitela
Boana albopunctata
Boana raniceps
Boana sibleszi
Boana boans
Boana pombali
Bokermannoyla circumdata
Aplastodiscus albosignata
Aplastodiscu weygoldti
Aplastodiscus albofrenatus
Nesorohyla kanaima
Callimedusa vaillanti
77
80
56
97
94
65
51
73
84
71
61
55
95
B. gr. pulchella
B. gr. faber
B. gr. pellucens
B. gr. albopunctata
B. gr. punctata
B. gr. albopunctata
B. gr. semilineata
0.020
Callimedusa vaillanti
Nesorohyla kanaima
Boana albomarginata
Boana balzani
Boana marianitae
Boana melanopleura
Boana buriti
Boana stenocephala
Boana jaguariaivensis
Boana leptolineata
Boana caingua
Boana cordobae
Boana goiana
Boana guentheri
Boana pulchella
Boana poaju
Boana riojana
Boana cambui
Boana cipoensis
Boana curupi
Boana polytaenia
Boana semiguttata
Boana rufitela
Boana albopunctata
Boana raniceps
Boana boans
Boana pombali
Boana sibleszi
Aplastodiscu weygoldti
Aplastodiscus albofrenatus
Aplastodiscus albosignatus
Bokermannoyla circumdata
SIAH1
1
0.61
0.7
1
0.77
0.51
0.95
1
0.7
0.98
0.87
0.66
0.78
1
0.52
0.74
1
B. gr. faber
B. gr. pulchella
B. gr. pellucens
B. gr. albopunctata
B. gr. semilineata
B. gr. punctata
0.006

e.

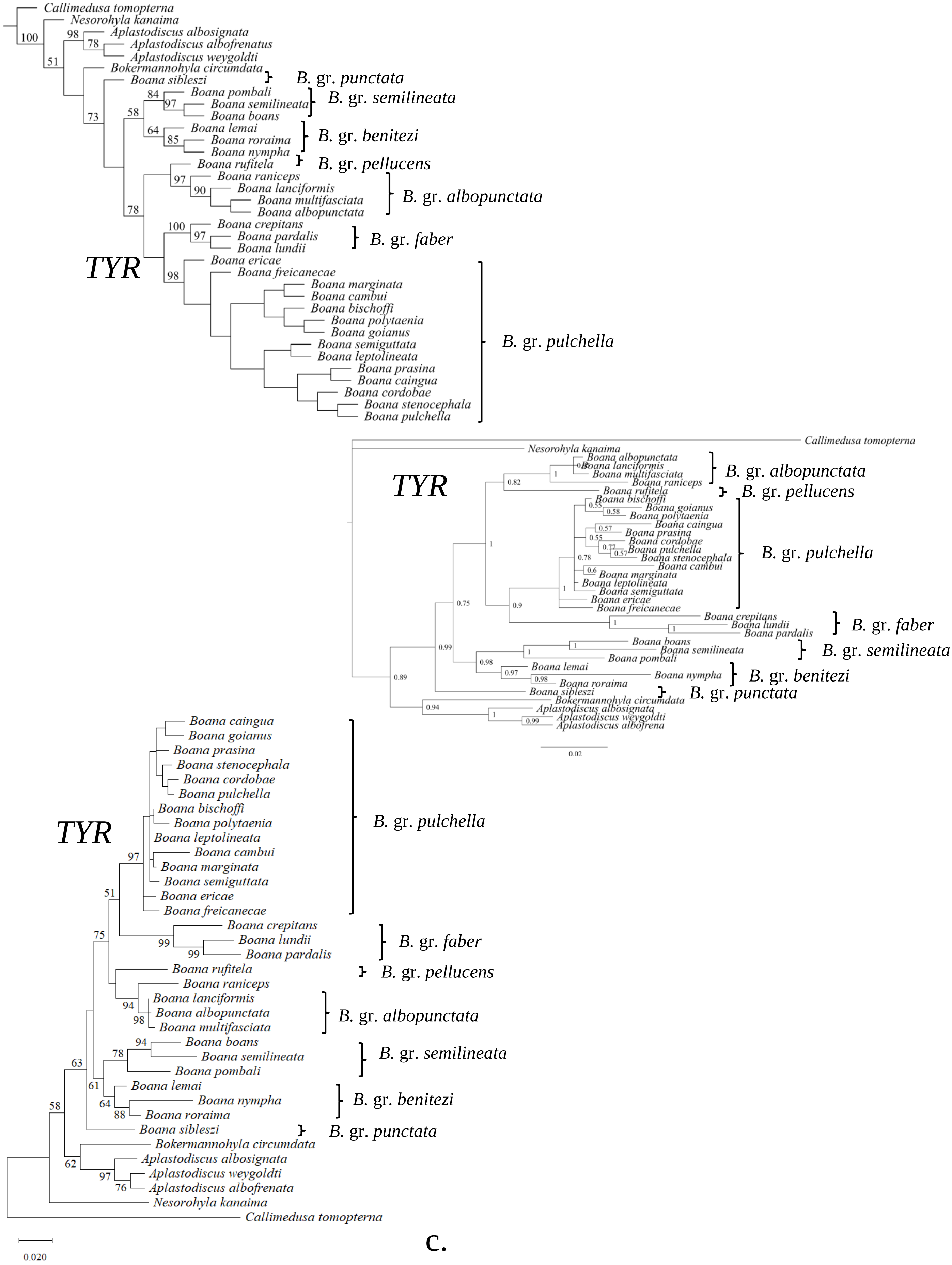
TYR
Callimedusa tomopterna
Nesorohyla kanaima
Aplastodiscus albosignata
Aplastodiscus albofrenatus
Aplastodiscus weygoldti
Bokermannohyla circumdata
Boana sibleszi
B. gr. punctata
Boana pombali
Boana semilineata
Boana boans
B. gr. semilineata
Boana lemai
Boana roraima
Boana nympha
B. gr. benitezi
Boana rufitela
B. gr. pellucens
Boana raniceps
Boana lanciformis
Boana multifasciata
Boana albopunctata
B. gr. albopunctata
Boana crepitans
Boana pardalis
Boana lundii
B. gr. faber
Boana ericae
Boana freicanecae
Boana marginata
Boana cambui
Boana bischoffi
Boana polytaenia
Boana goianus
Boana semiguttata
Boana leptolineata
Boana prasina
Boana caingua
Boana cordobae
Boana stenocephala
Boana pulchella
B. gr. pulchella
TYR
0.02
TYR
Aplastodiscus albofrenata
0.020

C.

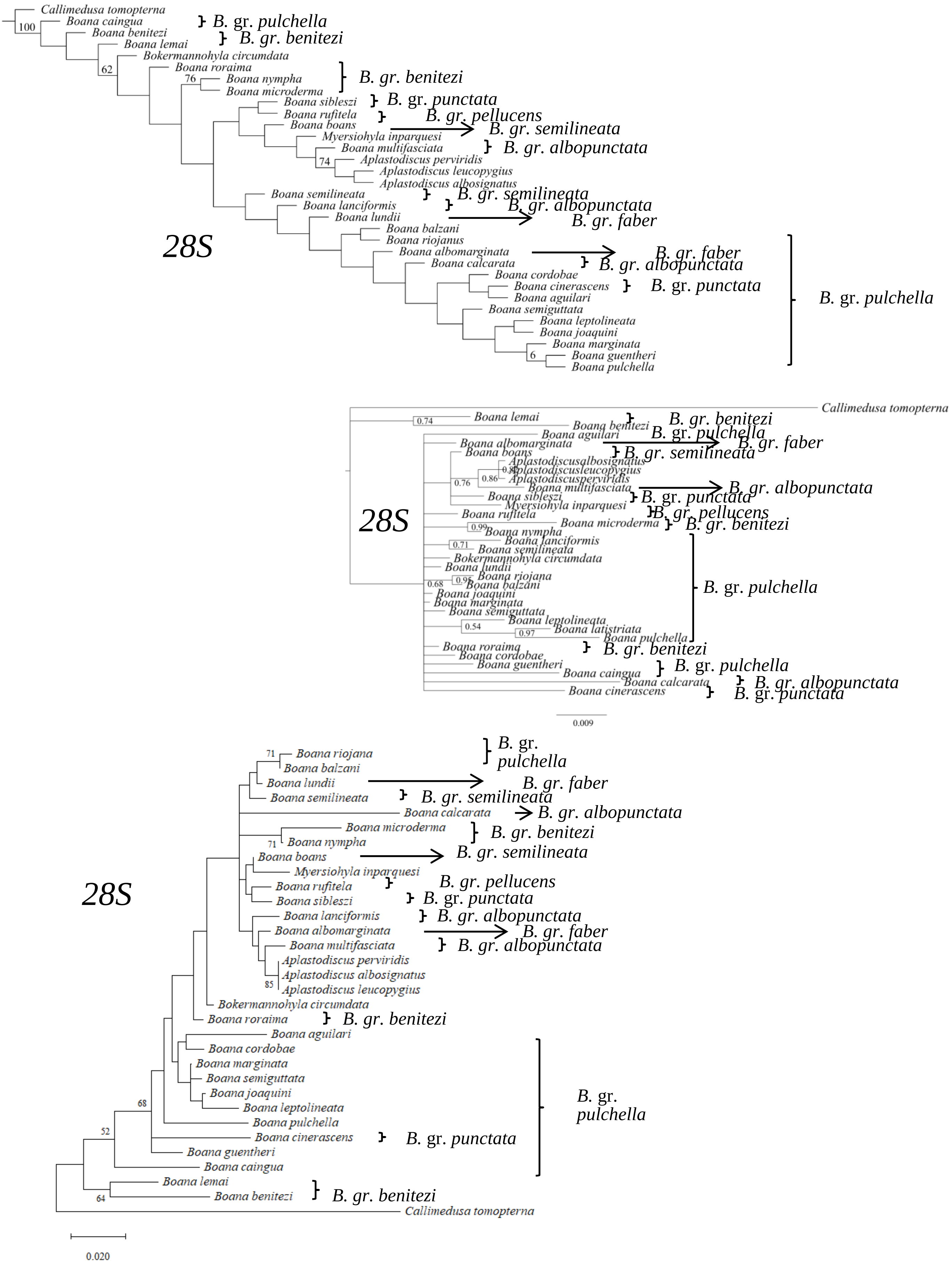
28S
Callimedusa tomopterna
Boana caingua
B. gr. pulchella
Boana benitezi
Boana lemai
B. gr. benitezi
Bokermannohyla circumdata
Boana roraima
Boana nympha
Boana microderma
B. gr. benitezi
Boana sibleszi
B. gr. punctata
Boana rufitela
B. gr. pellucens
Boana boans
B. gr. semilineata
Myersiohyla inparquesi
Boana multifasciata
B. gr. albopunctata
Aplastodiscus perviridis
Aplastodiscus leucopygius
Aplastodiscus albosignatus
Boana semilineata
B. gr. semilineata
Boana lanciformis
B. gr. albopunctata
Boana lundii
B. gr. faber
Boana balzani
Boana riojanus
Boana albomarginata
B. gr. faber
Boana calcarata
B. gr. albopunctata
Boana cordobae
Boana cinerascens
B. gr. punctata
Boana aguilari
Boana semiguttata
Boana leptolineata
Boana joaquini
Boana marginata
Boana guentheri
Boana pulchella
B. gr. pulchella
100
62
76
74
6
28S
Callimedusa tomopterna
Boana lemai
Boana benitezi
B. gr. benitezi
Boana aguilari
B. gr. pulchella
Boana albomarginata
B. gr. faber
Boana boans
B. gr. semilineata
Boana multifasciata
B. gr. albopunctata
Boana sibleszi
B. gr. punctata
Myersiohyla inparquesi
Boana rufitela
B. gr. pellucens
Boana microderma
B. gr. benitezi
Boana nympha
Boana lanciformis
Boana semilineata
Bokermannohyla circumdata
Boana lundii
Boana riojana
Boana balzani
Boana joaquini
Boana marginata
Boana semiguttata
Boana leptolineata
Boana latistriata
Boana pulchella
B. gr. pulchella
Boana roraima
B. gr. benitezi
Boana cordobae
Boana guentheri
Boana caingua
B. gr. pulchella
Boana calcarata
B. gr. albopunctata
Boana cinerascens
B. gr. punctata
0.74
0.76
0.86
0.99
0.71
0.68
0.95
0.54
0.97
0.009
28S
Boana riojana
Boana balzani
B. gr. pulchella
Boana lundii
B. gr. faber
Boana semilineata
B. gr. semilineata
Boana calcarata
B. gr. albopunctata
Boana microderma
Boana nympha
B. gr. benitezi
Boana boans
B. gr. semilineata
Myersiohyla inparquesi
Boana rufitela
B. gr. pellucens
Boana sibleszi
B. gr. punctata
Boana lanciformis
B. gr. albopunctata
Boana albomarginata
B. gr. faber
Boana multifasciata
B. gr. albopunctata
Aplastodiscus perviridis
Aplastodiscus albosignatus
Aplastodiscus leucopygius
Bokermannohyla circumdata
Boana roraima
B. gr. benitezi
Boana aguilari
Boana cordobae
Boana marginata
Boana semiguttata
Boana joaquini
Boana leptolineata
Boana pulchella
Boana cinerascens
B. gr. punctata
B. gr. pulchella
Boana guentheri
Boana caingua
Boana lemai
Boana benitezi
B. gr. benitezi
Callimedusa tomopterna
71
71
85
68
52
64
0.020

g.

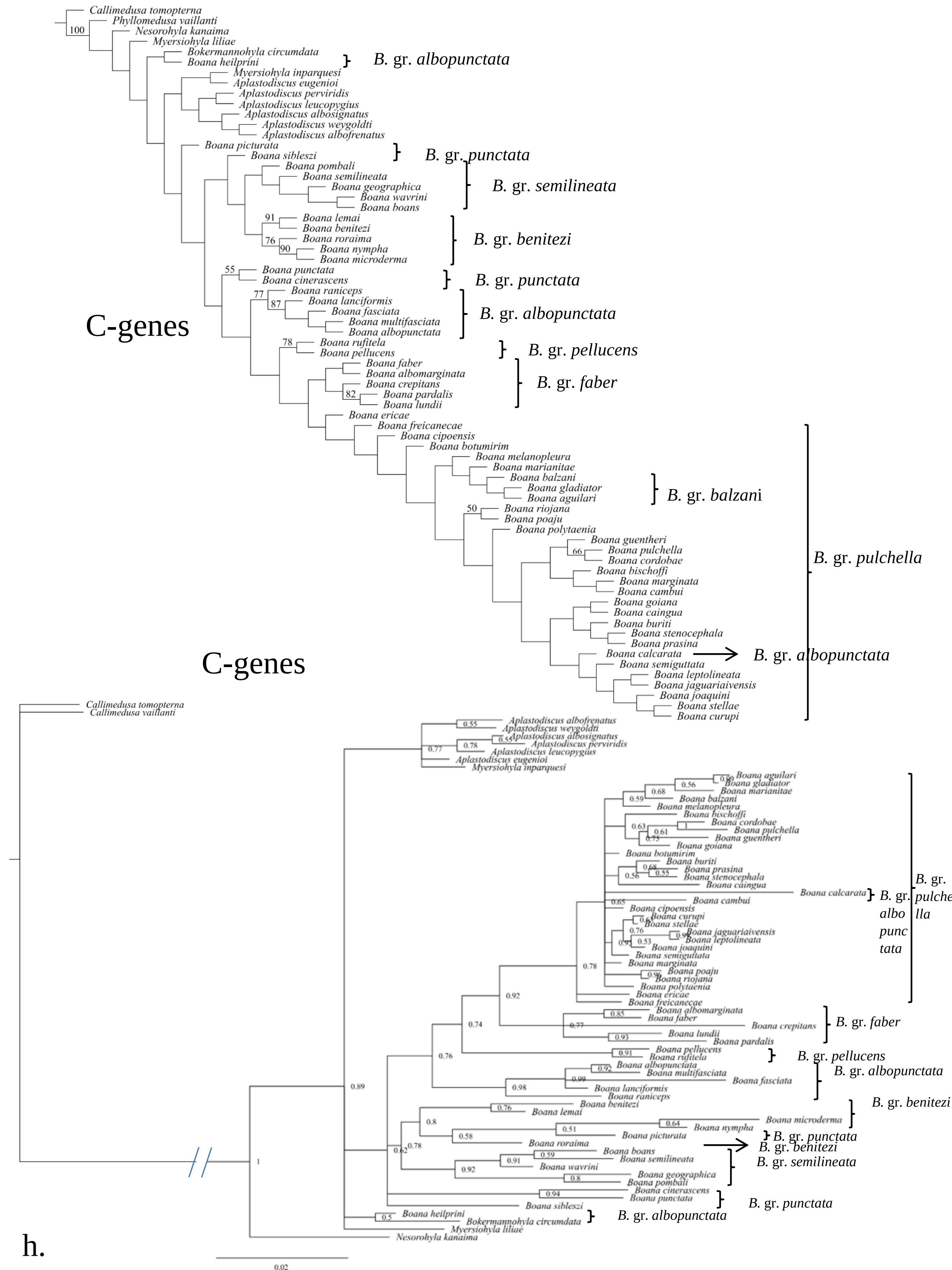
C-genes
Callimedusa tomopterna
Phyllomedusa vaillanti
100
Nesorohyla kanaima
Myersiohyla liliae
Bokermannohyla circumdata
Boana heilprini
B. gr. albopunctata
Myersiohyla inparquesi
Aplastodiscus eugenioi
Aplastodiscus perviridis
Aplastodiscus leucopygius
Aplastodiscus albosignatus
Aplastodiscus weygoldti
Aplastodiscus albofrenatus
Boana picturata
Boana sibleszi
B. gr. punctata
Boana pombali
Boana semilineata
Boana geographica
Boana wavrini
Boana boans
B. gr. semilineata
91
Boana lemai
Boana benitezi
76
Boana roraima
90
Boana nympha
Boana microderma
B. gr. benitezi
55
Boana punctata
Boana cinerascens
B. gr. punctata
77
Boana raniceps
87
Boana lanciformis
Boana fasciata
Boana multifasciata
Boana albopunctata
B. gr. albopunctata
78
Boana rufitela
Boana pellucens
B. gr. pellucens
Boana faber
Boana albomarginata
Boana crepitans
82
Boana pardalis
Boana lundii
B. gr. faber
Boana ericae
Boana freicanecae
Boana cipoensis
Boana botumirim
Boana melanopleura
Boana marianitae
Boana balzani
Boana gladiator
Boana aguilari
B. gr. balzani
50
Boana riojana
Boana poaju
Boana polytaenia
Boana guentheri
66
Boana pulchella
Boana cordobae
Boana bischoffi
Boana marginata
Boana cambui
Boana goiana
Boana caingua
Boana buriti
Boana stenocephala
Boana prasina
Boana calcarata
B. gr. albopunctata
Boana semiguttata
Boana leptolineata
Boana jaguariaivensis
Boana joaquini
Boana stellae
Boana curupi
B. gr. pulchella
C-genes
Callimedusa tomopterna
Callimedusa vaillanti
Aplastodiscus albofrenatus
Aplastodiscus weygoldti
Aplastodiscus albosignatus
Aplastodiscus perviridis
Aplastodiscus leucopygius
Aplastodiscus eugenioi
Myersiohyla inparquesi
Boana aguilari
Boana gladiator
Boana marianitae
Boana balzani
Boana melanopleura
Boana bischoffi
Boana cordobae
Boana pulchella
Boana guentheri
Boana goiana
Boana botumirim
Boana buriti
Boana prasina
Boana stenocephala
Boana caingua
Boana calcarata
B. gr. albo punc tata
B. gr. pulche lla
Boana cambui
Boana cipoensis
Boana curupi
Boana stellae
Boana jaguariaivensis
Boana leptolineata
Boana joaquini
Boana semiguttata
Boana marginata
Boana poaju
Boana riojana
Boana polytaenia
Boana ericae
Boana freicanecae
Boana albomarginata
Boana faber
Boana crepitans
Boana lundii
Boana pardalis
B. gr. faber
Boana pellucens
Boana rufitela
B. gr. pellucens
Boana albopunctata
Boana multifasciata
Boana fasciata
Boana lanciformis
Boana raniceps
B. gr. albopunctata
Boana benitezi
Boana lemai
B. gr. benitezi
Boana microderma
Boana nympha
Boana picturata
B. gr. punctata
Boana roraima
B. gr. benitezi
Boana boans
Boana semilineata
Boana wavrini
Boana geographica
Boana pombali
B. gr. semilineata
Boana cinerascens
Boana punctata
Boana sibleszi
B. gr. punctata
Boana heilprini
Bokermannohyla circumdata
B. gr. albopunctata
Myersiohyla liliae
Nesorohyla kanaima
0.02
h.

h.

C-genes

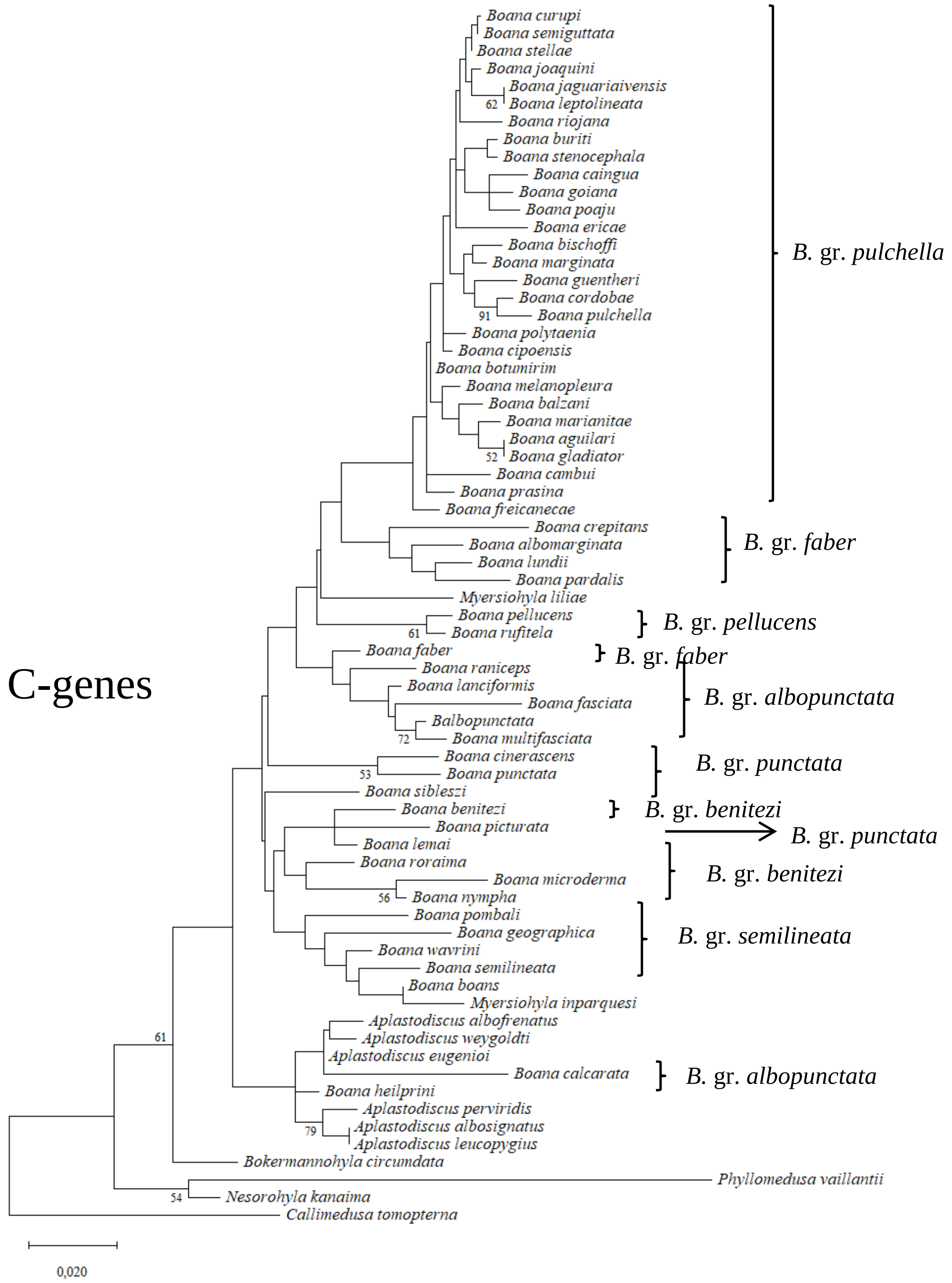

Supplementary Figure S1. Appendix 2. Phylogenetic trees corresponding to the studied markers (a. *CXCR4*, b. *FGBI7*, c. *RAG-1*, d. *RHO*, e. *TYR* f. *SIAH1*, g. *28S* and C-genes), and the methods used–MP, MB, and ML, corresponding to the 1st, 2nd, and 3rd trees for each marker, respectively). For Jackknife support values from the MP method, and bootstrap support values for the ML method, values below 50% were not presented.

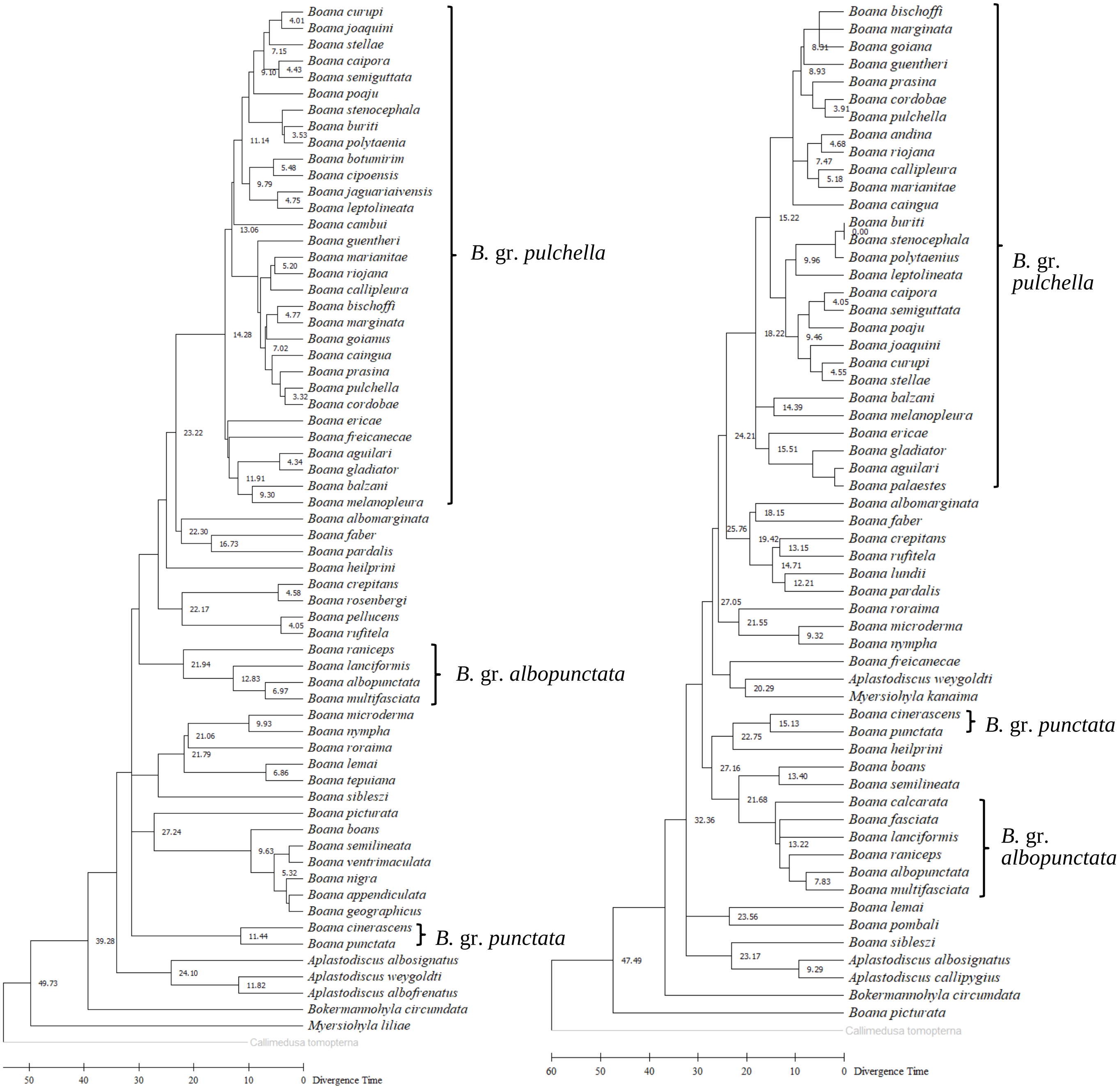
Boana curupi
Boana joaquini
Boana stellae
Boana caipora
Boana semiguttata
Boana poaju
Boana stenocephala
Boana buriti
Boana polytaenia
Boana botumirim
Boana cipoensis
Boana jaguariaivensis
Boana leptolineata
Boana cambui
Boana guentheri
Boana marianitae
Boana riojana
Boana callipleura
Boana bischoffi
Boana marginata
Boana goianus
Boana caingua
Boana prasina
Boana pulchella
Boana cordobae
Boana ericae
Boana freicanecae
Boana aguilari
Boana gladiator
Boana balzani
Boana melanopleura
Boana albomarginata
Boana faber
Boana pardalis
Boana heilprini
Boana crepitans
Boana rosenbergi
Boana pellucens
Boana rufitela
Boana raniceps
Boana lanciformis
Boana albopunctata
Boana multifasciata
Boana microderma
Boana nympha
Boana roraima
Boana lemai
Boana tepuiana
Boana sibleszi
Boana picturata
Boana boans
Boana semilineata
Boana ventrimaculata
Boana nigra
Boana appendiculata
Boana geographicus
Boana cinerascens
Boana punctata
Aplastodiscus albosignatus
Aplastodiscus weygoldti
Aplastodiscus albofrenatus
Bokermannohyla circumdata
Myersiohyla liliae
Callimedusa tomopterna
B. gr. pulchella
B. gr. albopunctata
B. gr. punctata
4.01
7.15
9.10
4.43
3.53
11.14
5.48
9.79
4.75
13.06
5.20
4.77
14.28
7.02
3.32
23.22
4.34
11.91
9.30
22.30
16.73
4.58
22.17
4.05
21.94
12.83
6.97
9.93
21.06
21.79
6.86
27.24
9.63
5.32
39.28
11.44
49.73
24.10
11.82
50 40 30 20 10 0 Divergence Time
Boana bischoffi
Boana marginata
Boana goiana
Boana guentheri
Boana prasina
Boana cordobae
Boana pulchella
Boana andina
Boana riojana
Boana callipleura
Boana marianitae
Boana caingua
Boana buriti
Boana stenocephala
Boana polytaenius
Boana leptolineata
Boana caipora
Boana semiguttata
Boana poaju
Boana joaquini
Boana curupi
Boana stellae
Boana balzani
Boana melanopleura
Boana ericae
Boana gladiator
Boana aguilari
Boana palaestes
Boana albomarginata
Boana faber
Boana crepitans
Boana rufitela
Boana lundii
Boana pardalis
Boana roraima
Boana microderma
Boana nympha
Boana freicanecae
Aplastodiscus weygoldti
Myersiohyla kanaima
Boana cinerascens
Boana punctata
Boana heilprini
Boana boans
Boana semilineata
Boana calcarata
Boana fasciata
Boana lanciformis
Boana raniceps
Boana albopunctata
Boana multifasciata
Boana lemai
Boana pombali
Boana sibleszi
Aplastodiscus albosignatus
Aplastodiscus callipygius
Bokermannohyla circumdata
Boana picturata
Callimedusa tomopterna
B. gr. pulchella
B. gr. punctata
B. gr. albopunctata
8.31
8.93
3.91
4.68
7.47
5.18
15.22
0.00
9.96
4.05
18.22
9.46
4.55
14.39
24.21
15.51
18.15
25.76
19.42
13.15
14.71
12.21
27.05
21.55
9.32
20.29
15.13
22.75
27.16
13.40
21.68
32.36
13.22
7.83
23.56
47.49
23.17
9.29
60 50 40 30 20 10 0 Divergence Time

a. b.

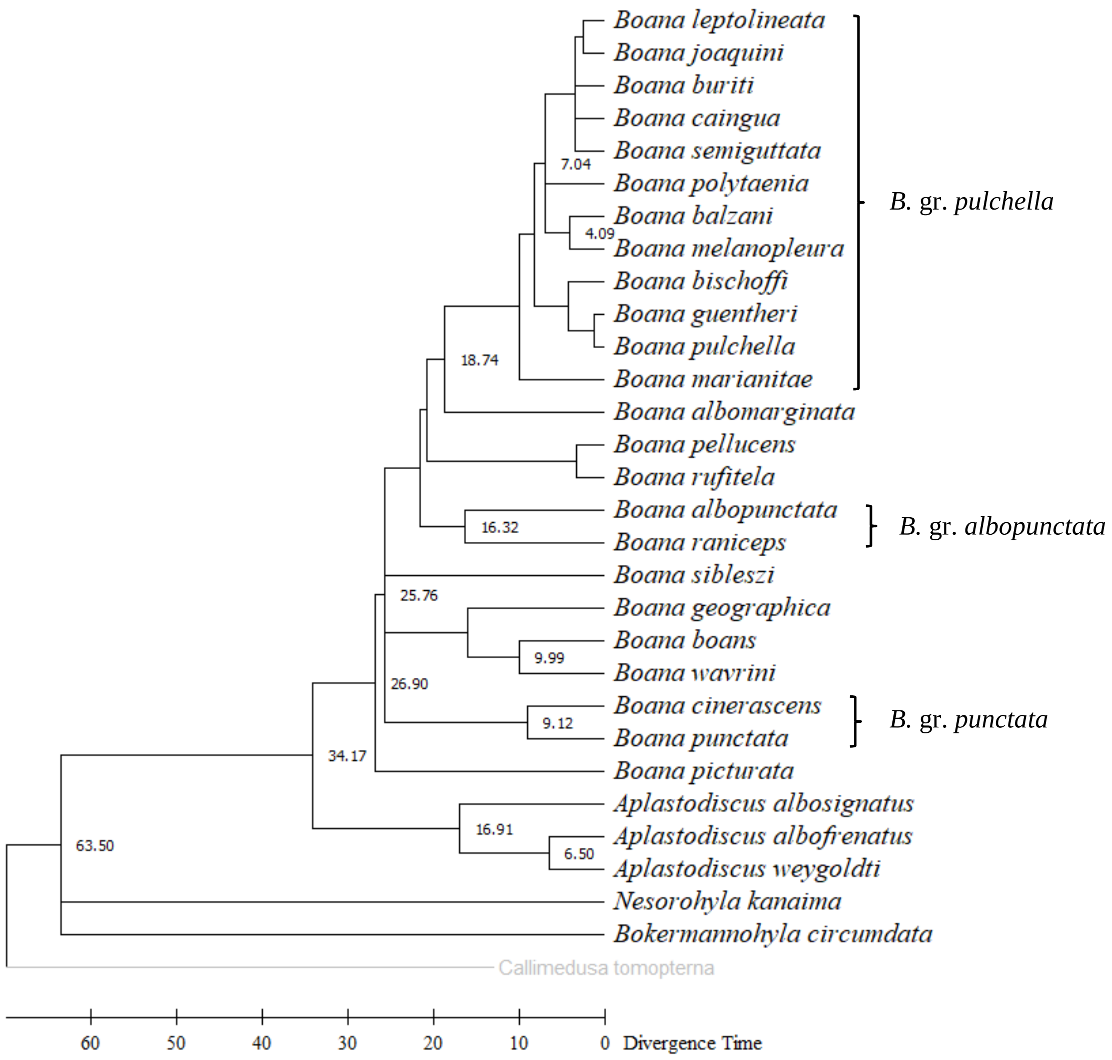

c.

Figure S2. Molecular dating of gene trees. a. *ND1*. b. *CYTB*, and c. *FGBI7* using the RelTime method in MEGA X.
